# Identity of the Silyl Ligand in an Iron Silyl Complex Influences Olefin Hydrogenation: An Experimental and Computational Study

**DOI:** 10.1021/acs.inorgchem.4c02533

**Published:** 2024-09-10

**Authors:** Daniel C. Najera, Marconi N. Peñas-Defrutos, Max García-Melchor, Alison R. Fout

**Affiliations:** † School of Chemical Sciences, University of Illinois at Urbana-Champaign, Urbana, Illinois 61801, United States; ‡ School of Chemistry, CRANN and AMBER Research Centres, Trinity College Dublin, College Green, Dublin 2, Ireland; § IU CINQUIMA/Química Inorgánica, Facultad de Ciencias, Universidad DeValladolid, Valladolid 47071, Spain; ∥ Department of Chemistry, Texas A&M University, College Station, Texas 77840, United States

## Abstract

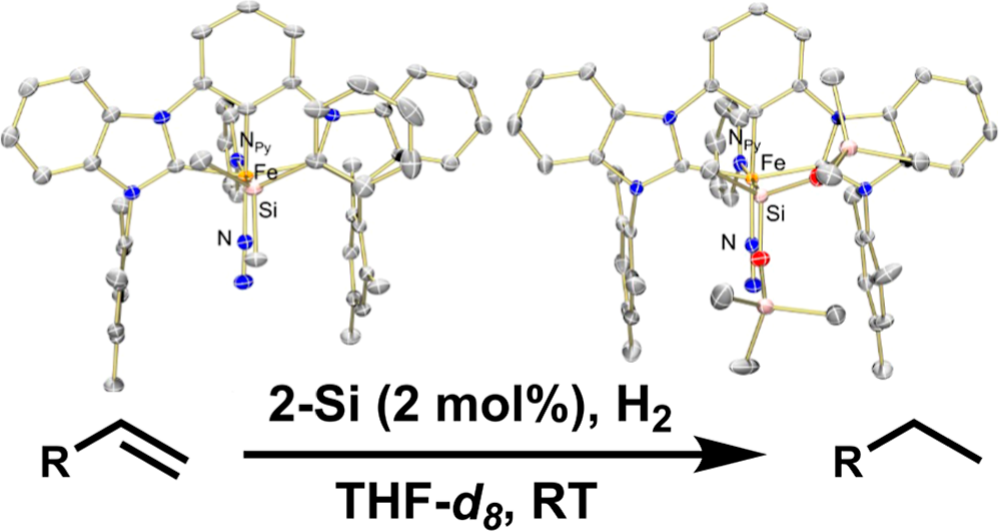

In this study, we explore the selective synthesis of iron silyl complexes using the reaction of an iron mesityl complex (^Mes^CCC)FeMes(Py) with various hydrosilanes. These resulting iron silyl complexes, (^Mes^CCC)Fe(SiH_2_Ph)(Py)(N_2_), (^Mes^CCC)Fe(SiMe_2_Ph)(Py)(N_2_), and (^Mes^CCC)Fe[SiMe(OSiMe_3_)_2_](Py)(N_2_), serve as effective precatalysts for olefin hydrogenation. The key to their efficiency in catalysis lies in the specific nature of the silyl ligand attached to the iron center. Experimental observations, supported by density functional theory (DFT) simulations, reveal that the catalytic performance correlates with the relative stability of dihydrogen and hydride species associated with each iron silyl complex. The stability of these intermediates is crucial for efficient hydrogen transfer during the catalytic cycle. The DFT simulations help to quantify these stability factors, showing a direct relationship between the silyl ligand’s electronic and steric properties and the overall catalytic activity. Complexes with certain silyl ligands exhibit better performance due to the optimal balance between the stability and reactivity of the key active catalyst. This work highlights the importance of ligand design in the development of iron-based hydrogenation catalysts.

## Abstract 2

This study investigates the synthesis of iron silyl complexes from an iron mesityl precursor. The resulting complexes, effective precatalysts for olefin hydrogenation, show that the silyl ligand’s electronic and steric properties significantly impact catalytic efficiency. Density functional theory simulations reveal that the stability of the dihydrogen and hydride intermediates correlates with catalytic performance, underscoring the importance of ligand design in optimizing iron-based hydrogenation catalysts.

## Introduction

**Figure 1:**
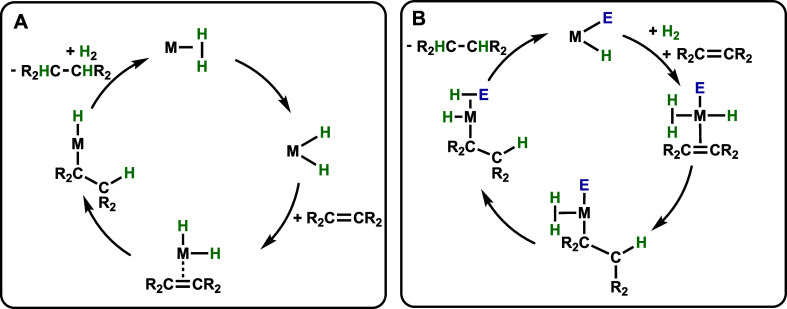
(A) Typical olefin hydrogenation pathway. (B) σ-CAM mechanism for olefin hydrogenation.

Catalytic hydrogenation is a critical component in the production of fine chemicals on an industrial scale.^1^ The activation of H_2_ is a key step in hydrogenation catalysis, which commonly proceeds via oxidative addition to a second- or third-row transition metal center (Figure 1a).^2^ Heterolytic cleavage of H_2_ through metal–ligand cooperativity provides an alternative route to achieve catalytic hydrogenation in systems where a formal oxidative addition process may not be readily accessible such as first-row transition metals.^3,4^ In this context, Nagashima et al. have demonstrated the aptitude of iron silyl complexes bearing 1,2-bis(dimethylsilyl)benzene ligands to facilitate the hydrogenation of olefins with various degrees of substitution.^5−7^ The redox-neutral activation of H_2_ proceeds via a σ-CAM (complex assisted metathesis) mechanism (Figure 1b) to generate an η^2^-silane iron hydride intermediate that can then undergo migratory insertion with the bound substrate. Hydrogen transfer from the η^2^-silane releases the hydrogenated product and regenerates the disilyl ligand.^5,8^ While this system relies on disilyl ligands, only one of the moieties participates in σ-CAM, suggesting that a system featuring a single silyl ligand could also engage in this reactivity and facilitate the incorporation of other ligands. Tonzetich et al. recently reported a (^Cy^PNP)FeSiR_3_L [^Cy^PNP = anion of 2,5-bis(dicylcohexylphosphinomethyl)pyrrole; L = N_2_ or PMe_2_Ph] catalyst that proceeds via a peripheral mechanism^9^ that permits σ-bond metathesis in the outer sphere of the Fe center.^10^

**Scheme 1:**
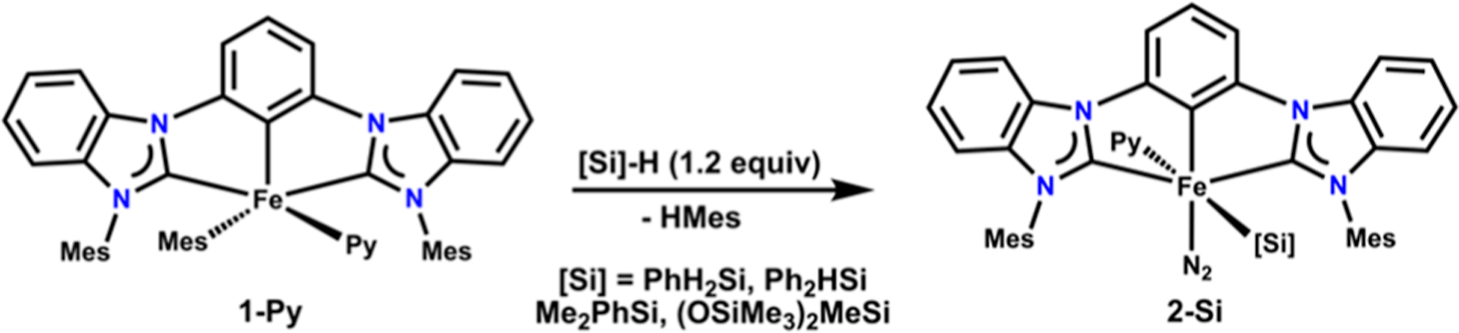
Synthesis of Iron(II) Silyl Complexes (**2-Si**) from the Activation of Silanes by **1-Py**

In our investigation of the chemistry of first-row transition metal complexes featuring strongly donating, monoanionic, bis(NHC) pincer CCC ligands, we recently succeeded in installing iron into the ^Mes^CCC platform [^Mes^CCC = bis(2,4,6-trimethylphenylbenzimidazol-2-ylidene)phenyl] to furnish a family of Fe(II) complexes (^Mes^CCC)FeMes(L) (**1L**) [L = pyridine (Py), 3,5-lutidine, PPh_3_, PMe_3_, MeCN, N_2_, CO; Mes = mesityl] that featured agostic interactions between the Mes ligand and the iron center.^9^ We envisioned that the anionic aryl donor in **1-Py** could promote the reaction with hydrosilanes to furnish isolable silyl complexes driven by the extrusion of mesitylene, a well-precedented approach both in our iron system and others.^12−17^ Herein, the synthesis and characterization of a family of iron(II) silyl complexes from the activation of various silanes by **1-Py** is described (Scheme 1). Compounds **1L** were effective in the catalytic hydrogenation of olefins, and a correlation between the hydrogenation activity and the identity of the silyl ligand was investigated and identified.

## Results and Discussion

Initial investigations into the reactivity with silanes focused on the activation of Ph_2_SiH_2_ by **1**-**Py**, since this reactivity was reported in other systems featuring anionic carbon donor ligands.^14,15,18^ The addition of 1.2 equiv of Ph_2_SiH_2_ to **1-Py** resulted in an immediate color change from deep purple to orange–red and the isolation of an orange solid after workup (Scheme 1). Characterization by ^1^H NMR spectroscopy revealed a spectrum consistent with a diamagnetic, C_s_-symmetric iron(II) complex (Figure S4). The three singlets at 2.32, 2.09, and 0.69 ppm, integrating to 6H each, were assigned to the methyl groups of the flanking mesityl moieties on the CCC scaffold. Protonation of the mesityl ligand in **1-Py** was evident from the disappearance of the upfield agostic resonance at −0.45 ppm in the ^1^H NMR spectrum and the observation of free mesitylene in the crude reaction mixture as assessed by the characteristic resonances in C_6_D_6_ at 2.16 and 6.72 ppm. The aryl region integrated to 30H, consistent with the CCC ligand backbone, the retention of pyridine, and two additional phenyl groups assigned to a bound diphenylsilyl moiety. A distinct singlet of the hydrogen atom of the diphenylsilyl ligand integrating to 1H was identified at 4.45 ppm and exhibited an upfield shift and a decreased ^1^*J*_Si–H_ coupling constant of 82.4 Hz relative to those of free Ph_2_SiH_2_ (Ph_2_SiH_2_ δ = 5.03 ppm, ^1^*J*_Si–H_ = 99 Hz in C_6_D_6_), collected from a commercial sample. The *ipso* carbon of the NHC donors was identified in the ^13^C{^1^H} NMR spectrum at 224.47 ppm, and the chemical shift of the silyl ligand in the ^29^Si{^1^H} NMR spectrum was located at 28.2 ppm. Additionally, an absorption at 2070 cm^–1^ in the ATR-IR spectrum is consistent with the presence of a dinitrogen ligand. On the basis of this spectroscopic data, the product was formulated as (^Mes^CCC)Fe(SiHPh_2_)(Py)(N_2_) (**2-SiHPh**_**2**_), and isolated in excellent yield (97%).

**Figure 2:**
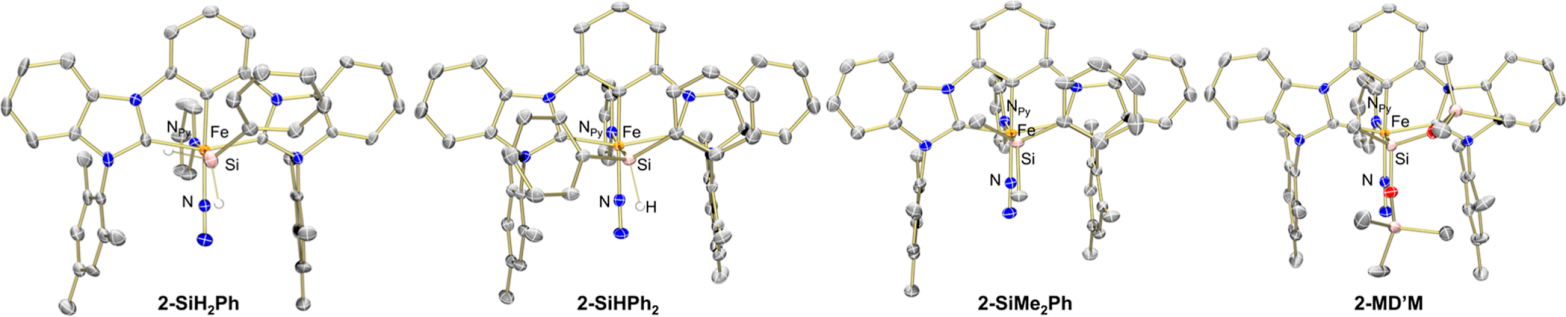
Molecular structures of **2-Si** with 50% probability ellipsoids. Solvent molecules and hydrogen atoms, except those bound to silicon, have been omitted for clarity.

Further characterization of **2-SiHPh**_**2**_ via single-crystal X-ray diffraction revealed a formally iron(II) center in an octahedral geometric arrangement of the CCC, pyridine, dinitrogen, and silyl ligands (Figure 2). The silyl and pyridine ligands are bound to iron in a trans configuration, while N_2_ occupies the position opposite the anionic aryl carbon of the CCC ligand. The positioning of N_2_ is somewhat unusual for late first-row transition metal complexes featuring CCC and pyridine ligands, where a general trend of pyridine binding in a coplanar fashion with the CCC framework has been upheld until now.^19^ Density functional theory (DFT) calculations at the ωB97-XD level (see Supporting Information for details) confirmed that pyridine bound where N_2_ is to be disfavored by ca. +7.5 kcal/mol (Figure S27).

Structural parameters of the ligand show a Fe–C_Ar_ distance of 1.898(3) Å and Fe–NHC distances of 1.932(3) and 1.925(3) Å that are consistent with other iron(II) complexes with this ligand.^9^ The dinitrogen ligand N–N distance of 1.112(4) Å and nearly linear Fe–N–N angle of 175.5(3), along with the observed N–N stretch in the IR spectrum (i.e., 2070 cm^–1^), indicate little activation compared to free N_2_ (N–N = 1.0977 Å and υ_NN_ = 2331 cm^–1^).^20^ The Fe–Si bond length of 2.3272(9) Å is within the range of those in iron(II) PNP complexes, (^Cy^PNP)Fe(SiHPh_2_) and (^tBu^PNP)Fe(SiH_2_Ph) [^Cy^PNP = bis(dicyclohexylphosphinomethyl)pyrrole], of 2.384(1) and 2.2680(12) Å, respectively.^14^

The scope of this reaction was investigated via the synthesis and characterization of a family of iron silyl complexes. Slight modification of the previously described protocol confirmed this process to be amenable to primary (PhSiH_3_) and tertiary silanes (Me_2_PhSiH, MD’M) (MD’M = 1,1,1,3,5,5,5-heptamethyltrisiloxane), furnishing the family of iron(II) silyl complexes **2-Si**: (^Mes^CCC)Fe(SiH_2_Ph)(Py)(N_2_) (**2-SiH**_**2**_**Ph**), (^Mes^CCC)Fe(SiMe_2_Ph)(Py)(N_2_) (**2-SiMe**_**2**_**Ph**), and (^Mes^CCC)Fe(SiMe(OSiMe_3_)_2_)(Py)(N_2_) (**2-MD’M**), respectively (Scheme 1). In contrast, the reaction of **1-Py** with triethylsilane, triethoxysilane, and triphenylsilane did not produce the corresponding silyl complexes under the described conditions. The activation of the primary and secondary silanes in the family of complexes **2-Si** requires reaction times between 30 min and 10 h in the order **2-SiH**_**2**_**Ph** ≈ **2-SiHPh**_**2**_ < **2-MD’M** < **2-SiMe**_**2**_**Ph**. Characterization of these complexes by ^1^H NMR spectroscopy showed similar features (see Supporting Information). Additionally, the ATR-IR spectra are consistent with the presence of a dinitrogen ligand across all of the complexes. Spectroscopic features of this family of complexes **2-Si** are summarized in Table 1.

**Table 1:** Selected Spectroscopic Parameters of **2-Si**

complex	δ ^1^H_Si–H_ (ppm)	δ ^13^C_NHC_ (ppm)	δ ^29^Si_Fe_ (ppm)	ν_NN_ (cm^–1^)
**2-SiH**_**2**_**Ph**	3.96	223.69	10.87	2075
**2-SiHPh**_**2**_	4.45	224.47	28.22	2070
**2-SiMe**_**2**_**Ph**		225.52	21.83	2061
**2-MD’M**		233.44	28.98	2084

Structural characterization by single-crystal X-ray diffraction confirmed the analogous structures of **2-Si** (Figure 1). Comparison of the structural parameters of **2-Si** reveals little deviation between this family of complexes, precluding any significant changes in the electronic structure of the ligands (Table S2). The Fe–Si distances compare favorably with those observed in other iron(II) silyl complexes.^14,21,22^ Likewise, the dinitrogen ligands remain largely unactivated, as evidenced by the N–N distances and nearly linear Fe–N–N angles (Table S2).

The competency of **2-Si** in the hydrogenation of olefins was evaluated. Using styrene as a model substrate revealed that the hydrogenation to ethylbenzene catalyzed by **2-Si** (2 mol %) proceeded to varying degrees of completion according to the identity of the silyl ligand (Table 2, entries 1–4, Figure S20). Monitoring of the reaction over the course of 1 h showed **2-MD’M** and **2-SiMe**_**2**_**Ph** as the most efficient precatalysts, achieving full conversion to ethylbenzene (Table 2, entries 3–4). The primary and secondary silyl complexes **2-SiH**_**2**_**Ph** and **2-SiHPh**_**2**_ achieved 41 and 84% conversion, respectively, in the same time frame (Table 2, entries 1–2).

**Table 2:**
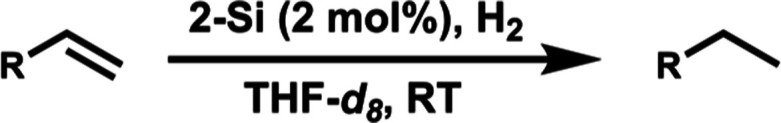
Olefin Hydrogenation by **2-Si**^a^

entry	substrate	precatalyst	time	conversion (%)
1	styrene	**2-SiH**_**2**_**Ph**	1 h	41
2		**2-SiHPh**_**2**_	1 h	84
3		**2-MD’M**	1 h	>99
4		**2-SiMe**_**2**_**Ph**	1 h	>99
5	1-octene	**2-SiH**_**2**_**Ph**	2 h	>99
6		**2-MD’M**	2 h	>99
7	3,3-dimethylbutene	**2-SiH**_**2**_**Ph**	40 h	45
8		**2-MD’M**	40 h	97
9	4-vinylcyclohexene^c^	**2-SiH**_**2**_**Ph**	2 h	>99
10		**2-MD’M**	2 h	>99
11		**2-MD’M**	20 min	34
12		**2-SiMe**_**2**_**Ph**	20 min	32
13^b^		**2-MD’M**	1 h	19
14^b^		**2-SiMe**_**2**_**Ph**	1 h	42

a Conversion determined by ^1^H NMR spectroscopy using mesitylene as an internal standard.

b All reactions were run at 4 atm of H2 pressure except entries 13 and 14, which were carried out at 1 atm.

c Hydrogenation only occurs at the terminal olefin.

Further comparison of the hydrogenation efficiency of **2-MD’M** and **2-SiH**_**2**_**Ph** showed full conversion of 1-octene (Table 2, entries 5–6). A more sterically demanding substrate, 3,3-dimethylbutene, once again highlighted the differences between the two precatalysts, achieving 97% conversion with **2-MD’M** and 45% conversion with **2-SiH**_**2**_**Ph** after 40 h (Table 2, entries 7–8, Figures S23 and S24). Catalysts **2-MD’M** and **2-SiH**_**2**_**Ph** showed full conversion of 4-vinylcyclohexene to 4-ethylcyclohexene after 2 h under 4 atm of H_2_ (Table 2, entries 9–10). The hydrogenation activities of the tertiary silyl complexes **2-MD’M** and **2-SiMe**_**2**_**Ph** were also found to be similar even at short reaction times. For example, 4-vinylcyclohexene showed respective conversions to 4-ethylcyclohexene after 20 min of 34 and 32% for **2-MD’M** and **2-SiMe**_**2**_**Ph** (Table 2, entries 11–12). Decreasing the H_2_ pressure to 1 atm led to a more pronounced difference with 42% (**2-SiMe**_**2**_**Ph**) and 19% (**2-MD’M**) conversion after 1 h (Table 2, entries 13–14). These results hint at a higher hydrogenation efficiency with **2-SiMe**_**2**_**Ph**, although the difference was not significant under normal reaction conditions (excess of H_2_).

To better understand the distinct hydrogenation activities among **2-Si**, reactivity studies were undertaken. Addition of 4 atm of H_2_ to **2-SiHPh**_**2**_ in C_6_D_6_ revealed a mixture of **2-SiHPh**_**2**_ and a new product with a characteristic broad resonance at around −3.68 ppm (Figures 2A and S18) in the ^1^H NMR spectrum. Significant overlap of the resonances precluded complete identification of those corresponding to the new complex, except for the appearance of three distinct mesityl singlets of the ligand at 1.98, 1.95, and 0.65 ppm, slightly shifted from those of the starting material. The Si–H resonance was assigned to the singlet at 3.45 ppm. Exposure of this mixture to a N_2_ atmosphere completely regenerated **2-SiHPh**_**2**_. Based on this data, the new complex was tentatively formulated as the dihydrogen complex (^Mes^CCC)Fe(SiHPh_2_)(H_2_)(Py) (**H**_**2**_**···2-SiHPh**_**2**_) arising from displacement of the N_2_ ligand by H_2_, with a similar chemical shift to that previously observed with cobalt.^21^ Full conversion to **H**_**2**_**···2-SiHPh**_**2**_ could not be achieved, but heating the reaction mixture to 65 °C increased the amplitude of the corresponding resonances in the ^1^H NMR spectrum.

**Figure 3:**
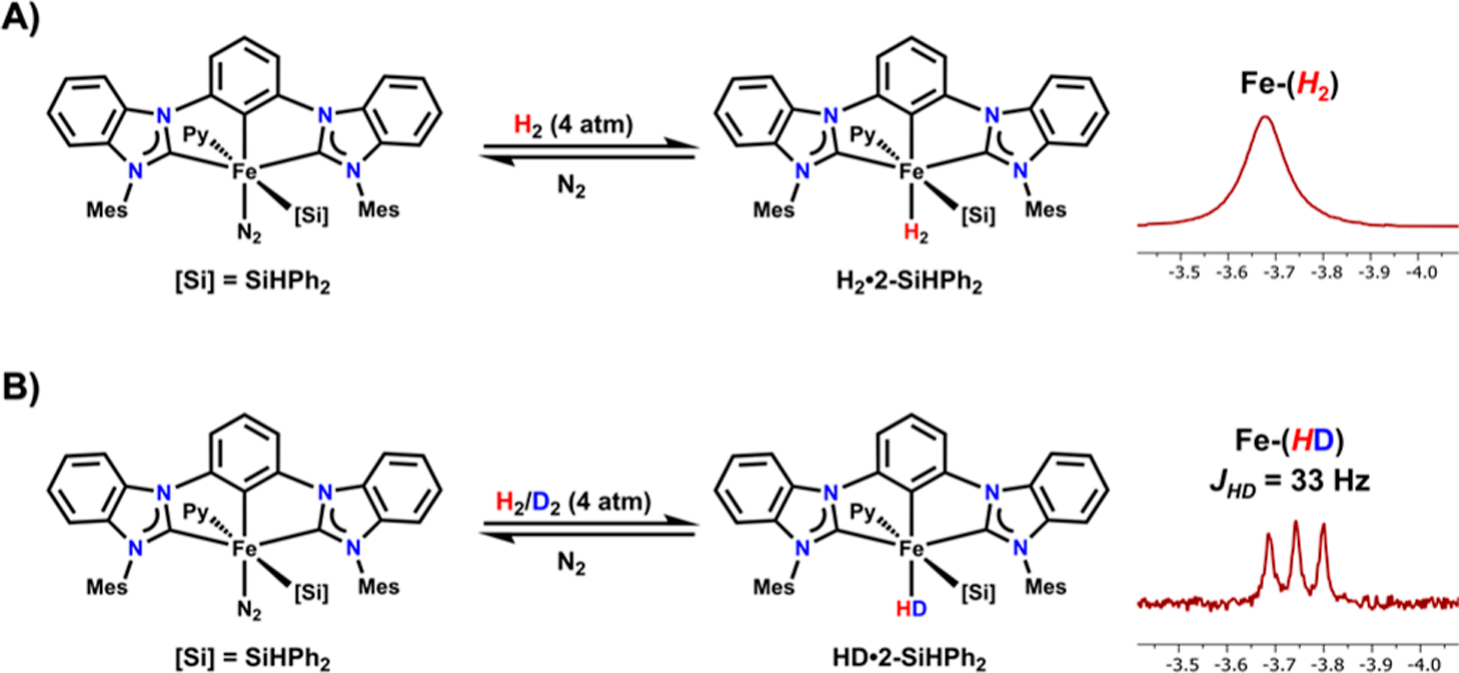
Formation and truncated ^1^H NMR spectra of the upfield region of (A) **H**_**2**_**···2-SiHPh**_**2**_ and (B) **HD···2-SiHPh**_**2**_.

To further support the assignment of **H**_**2**_**···2-SiHPh**_**2**_, deuteration experiments were conducted (Figure 3). The addition of 4 atm of D_2_ to **2-SiHPh**_**2**_ revealed an upfield resonance at −3.74 ppm in the ^2^H NMR spectrum, consistent with the expected deuterium-bound species (Figure S16). An additional resonance at 3.46 ppm indicated exchange between D_2_ and the Si–H bond, evidence of the cleavage of the H–H bond. The incorporation of deuterium could occur via σ-partner exchange between the bound D_2_ and the silyl ligand, forming a deuteride complex with an η^2^-Ph_2_SiHD ligand. A second σ-partner exchange event yielded the deuterated silyl complex and one equivalent of HD gas.

The cleavage of the H–H bond was probed by HD scrambling with **2-SiHPh**_**2**_ and a 1:1 mixture of H_2_ and D_2_. The ^1^H NMR spectrum of this reaction after 1 h showed the formation of HD gas as a triplet at 4.43 ppm (*J*_HD_ = 43 Hz), along with a singlet at 4.47 ppm assigned to H_2_, and a triplet at −3.74 ppm consistent with the formation of the HD-bound complex **HD···2-SiHPh**_**2**_ (Figures 2B and S18). The *J*_HD_ coupling constant of 33 Hz is identical to that observed in the dihydrogen Co complex (^Mes^CCC)Co(PPh_3_)(H_2_), corresponding to a *r*_HH_ distance of 0.87 Å as determined by the method by Morris et al.,^23,24^ further supporting the neutral binding mode of H_2_.

Having established the activation of H_2_ by **2-SiHPh**_**2**_, similar reactions with other **2-Si** complexes were pursued. Each of the silyl complexes exhibited resonances consistent with coordination of H_2_, with slightly different chemical shifts, consistent with minor differences in the magnetic environment caused by the silyl ligand. In the case of **2-SiH**_**2**_**Ph**, the dihydrogen resonance is broader than that in the other complexes, likely because of the exchange reaction with the two hydrogens in the silyl ligand. Addition of a 1:1 mixture of H_2_ and D_2_ to **2-SiH**_**2**_**Ph** supported this exchange by the rapid formation of HD gas (Figure S19), although coordination of HD was not observed.

**Figure 4:**
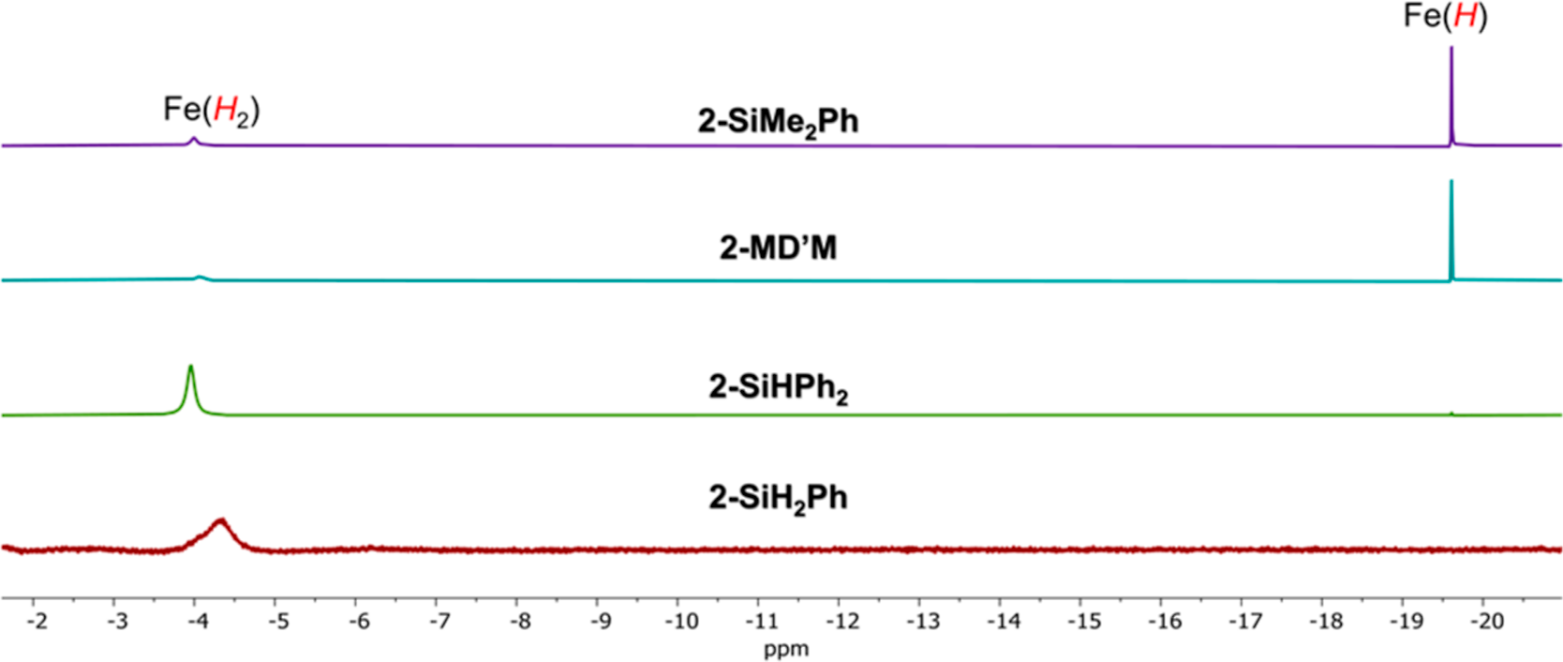
Truncated ^1^H NMR spectra of the upfield region of the reaction of **2-Si** with H_2_ in C_6_D_6_.

Reactions of H_2_ with the most active precatalysts, namely, **2-MD’M** or **2-SiMe**_**2**_**Ph** (Table 2), produced minimal amounts of the dihydrogen adduct. Instead, the major appearance of a new resonance further upfield at −19.64 ppm was observed in both cases (Figure 4). This chemical shift is similar to that found in the hydride complex (^DIPP^CCC)Fe(H)(Py)(N_2_)^25^ (^DIPP^CCC = bis(2,6-diisopropylphenylbenzimidazol-2-ylidene)phenyl) and the reported (^Mes^CCC)Fe(H)(Py)(N_2_) (**3**).^12^

Resonances corresponding to the free silane were also identified at 4.64 [(SiMe_3_O)_2_MeSiH] and 4.41 (Me_2_PhSiH) ppm (Figure S14). Based on this information, the new complex was assigned to a putative iron hydride complex **3** (^Mes^CCC)Fe(H)(Py)(N_2_). No HD scrambling was observed with these complexes, likely because the formation of **3** is more favorable than the reverse reaction.

The experimental results described thus far suggest a strong correlation between the relative stability of the Fe–H_2_ and Fe–H species and the catalytic performance summarized in Table 2. To further understand the distinct behaviors of the different Fe–Si compounds, we performed DFT calculations in implicit THF at the ωB97-XD level. First, we optimized the structures of the (^Mes^CCC)Fe(Si^1^R^2^R^3^)(Py)(H_2_) complexes (**H**_**2**_**···2-Si**) formed upon reacting compound **2** with H_2_, as shown in Figure S25.

**Figure 5:**
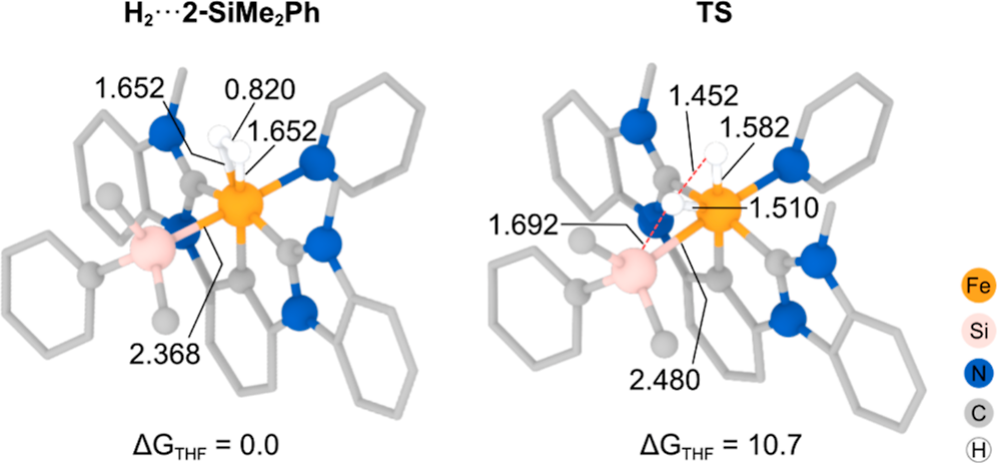
Optimized structures of the **H**_**2**_**···2-SiMe**_**2**_**Ph** intermediate (left) and the transition state associated with H–H bond cleavage (right). Most of the H atoms have been omitted for clarity, and the mesityl substituents of the ^Mes^CCC ligand have been simplified. The calculated relative Gibbs energies in THF solution, Δ*G*_THF_, are given in kcal/mol, and relevant distances are shown in Å.

Figure 5 (left) displays the structure of the dihydrogen adduct with a SiMe_2_Ph moiety (**H**_**2**_**···2-SiMe**_**2**_**Ph**). Notably, the Fe–Si bond distance remains largely unaffected by the substitution of N_2_ with H_2_ (i.e., 2.381 vs 2.368 Å). However, the coordination of the H_2_ molecule, which adopts an orthogonal orientation relative to the Si–Fe–Py plane, significantly weakens the H–H bond (i.e., 0.820 vs 0.743 Å in free H_2_), favoring the formation of symmetric Fe···H interactions at distances of 1.652 Å.^26^

The cleavage of the H–H bond from the **H**_**2**_**···2-Si** complexes leads to the formation of the respective silane and an iron hydride. Figure 5 (right) illustrates the transition state computed from **H**_**2**_**···2-SiMe**_**2**_**Ph**, wherein hydrogen migration involves remarkably short Fe–H distances, revealing a particularly strong interaction with the transiently bound H atom (1.510 Å) and a concomitant stretching of the Fe–Si bond (from 2.368 to 2.480 Å). This transition state has an associated activation barrier of 10.7 kcal/mol, which is easily surmountable at room temperature, resulting in the metastable hydride complex (^Mes^CCC)FeH(Py)(HSiMe_2_Ph) being formed in an endergonic process by +9.6 kcal/mol (Figure S26, left). This intermediate features the HSiMe_2_Ph group interacting weakly with the iron metal center (Fe–Si: 2.993 Å), allowing favorable substitution of the silane by N_2_ (by 13.8 kcal/mol) to form the proposed hydride complex **3** (Figure S26, right).

We next posited that the energetics associated with the conversion of **H**_**2**_**···2-Si** to the iron hydride complex **3** (labeled as Δ*G*_H_2_/H_) might determine the concentration of the active hydride species in solution. Indeed, we found that the trend in the computed Δ*G*_H_2_/H_ values, summarized in Table 3, accurately reflects the catalytic efficiency of the **2-Si** precatalysts observed experimentally (Table 2, reproduced in Table 3). For example, Table 2 (entries 1–4) clearly indicates that the poorest performance in styrene hydrogenation is obtained with **2-SiH**_**2**_**Ph** (41% conversion), which exhibits the most endergonic Δ*G*_H_2_/H_ value of +5.0 kcal/mol (Table 3). Incomplete transformation (84%) was also observed with **2-SiHPh**_**2**_ (Table 2, entry 2), for which DFT calculations predict a Δ*G*_H_2_/H_ value of +3.4 kcal/mol, indicating a more stable dihydrogen compound compared with the hydride, as observed experimentally (Figure 3). Conversely, the best precatalysts, namely, **2-MD’M** and **2-SiMe**_**2**_**Ph**, present lower Δ*G*_H_2_/H_ values of +1.2 and −4.2 kcal/mol, respectively, confirming that hydride formation is more favored in these cases, and consequently, hydrogenation is more efficient.

**Table 3:** Gibbs Energies (in kcal/mol) Calculated in THF for the Conversion of **H**_**2**_**···2-Si** to **3**, Δ*G*_H_2_/H_, with Different Silyl Groups, according to the Equation below^a^

silyl group	Δ*G*_H2/H_	styrene conversion (%)
**2-SiH**_**2**_**Ph**	+5.0	41
**2-SiHPh**_**2**_	+3.4	84
**2-MD’M**	+1.2	>99
**2-SiMe**_**2**_**Ph**	–4.2	>99

a Experimental conversions for styrene hydrogenation with the **H**_**2**_**···2-Si** complexes from Table 2 are also included for clarity.

However, the slightly endergonic transformation from **H**_**2**_**···2-MD’M** to **3**, with a Δ*G*_H_2_/H_ value of +1.2 kcal/mol, contrasts with the ^1^H NMR observations (Figure 3), which indicate predominant hydride formation for the SiMe(OSiMe_3_)_2_ compound. This discrepancy prompted us to re-examine the nature of hydride species **3** in solution. Further computational investigations revealed that the dimerization of **3** to form (μ-N_2_)[(^Mes^CCC)Fe(H)(Py)]_2_ (**4**) is thermodynamically favored by −8.7 kcal/mol (Figure S28). This finding is in line with the isolation of **4**, which was previously independently prepared and fully characterized.^12^ The catalytic activity of **4** was also confirmed to surpass that of the **2-Si** complexes.^12^ The superior catalyst activity of **4** compared to **2-Si** supports the role of the iron silyl complexes as precatalysts in hydrogenation catalysis.

Lastly, the difference observed in entries 13–14 in Table 2, showing comparatively higher activity of **2-SiMe**_**2**_**Ph** versus **2-MD’M**, particularly when working with reduced pressure of H_2_, may be attributed to (i) the more favorable N_2_ by H_2_ substitution leading to **H**_**2**_**···2-SiMe**_**2**_**Ph** (by 1.1 kcal/mol more accessible than its MD’M analogue) and (ii) the higher exergonicity of hydride formation from **H**_**2**_**···2-SiMe**_**2**_**Ph** (Δ*G*_H_2_/H_ = −4.2 kcal/mol vs +1.2 kcal/mol, see Table 3).

## Conclusions

In conclusion, (^Mes^CCC)Fe silyl complexes have been selectively synthesized from the reaction of (^Mes^CCC)FeMes(Py) with hydrosilanes. Our investigations demonstrate that some of these complexes are effective precatalysts in the hydrogenation of olefins and that the identity of the silyl ligand has a marked effect on the hydrogenation efficiency. The synergy of experimental results with DFT simulations allowed us to relate the catalytic performance to the relative stability of the dihydrogen and hydride species in each case, without neglecting the potentially limiting H_2_ coordination equilibria under reduced pressures of H_2_ gas.

This work highlights the crucial role of ligand design in optimizing catalytic performance and provides a deeper understanding of the mechanistic aspects of hydrogenation catalysis involving iron silyl complexes. The insights gained here could inform the development of more efficient and selective hydrogenation catalysts, potentially impacting various industrial processes that rely on catalytic hydrogenation.

## Experimental Section

### General Considerations

All manipulations of air- and moisture-sensitive compounds were carried out in the absence of water and dioxygen in an MBraun inert atmosphere glovebox under a dinitrogen atmosphere, except where specified otherwise. All glassware was oven-dried for a minimum of 8 h and cooled in an evacuated antechamber prior to use in the glovebox. Solvents for sensitive manipulations were dried and deoxygenated on a Glass Contour System (SG Water USA, Nashua, NH) and stored over 4 Å molecular sieves purchased from Strem following a literature procedure prior to use.^27^ The complex (^Mes^CCC)FeMes(Py) was prepared following the reported procedure.^11^ Benzene-*d*_6_ and THF-*d*_8_ were purchased from Cambridge Isotope Laboratories and were degassed and stored over 4 Å molecular sieves prior to use. Silane reagents were purchased from MilliporeSigma and stored over 4 Å molecular sieves. Celite 545 (J. T. Baker) was dried in a Schlenk flask for 24 h under dynamic vacuum while heating to at least 150 °C prior to use in a glovebox. No other uncommon hazards were noted.

NMR spectra were recorded at room temperature on a Bruker spectrometer operating at 600 MHz (^1^H) and 151 MHz (^13^C) and referenced to the residual HC_6_D_5_ and HC_4_D_7_O resonance (δ in parts per million and J in Hz). Solid-state infrared spectra were recorded using a PerkinElmer Frontier FT-IR spectrophotometer equipped with a KRS5 thallium bromide/iodide universal attenuated total reflectance accessory. X-ray crystallography was performed at the George L. Clark X-ray Facility at UIUC. Single-crystal X-ray diffraction data were collected with the use of multimirror monochromatized Mo Kα radiation (0.71073 Å) at 100 K on a Bruker D8 Venture diffractometer equipped with a Photon 100 detector. Combinations of 0.5° φ and ω scans were used to collect the data. The collection, cell refinement, and integration of intensity data were carried out with the APEX2 software.^28^ Multiscan absorption correction was performed using SADABS.^29^ The structures were solved with XT^30^ and refined with the full-matrix least-squares SHELXL^31^ program within the Olex2^32^ refinement GUI.

### Computational Methods

DFT calculations reported in this work were carried out using the dispersion corrected hybrid functional ωB97X-D^33^ and the Gaussian09 software.^34^ C, Si, and H atoms were described using the double-ζ basis set 6-31G(d,p), whereas the same basis set plus diffuse functions was employed to describe the more electronegative N and O atoms. The Fe metal was described using the effective core potential Lanl2dz,^35^ including a f-polarization function (exponent = 2.462).^36^ Fe(II) complexes were modeled as singlets, according to the experimental data.^11,12^ Geometry optimizations were performed in implicit THF using the SMD solvation model^37^ (ε = 7.43) without imposing any constraint and using the X-ray structures as initial guesses, when available.^38^

The nature of the stationary points was further verified through a vibrational frequency analysis. As expected, all of the energy minima were confirmed to display only real vibrational frequencies, whereas transition states exhibited one single imaginary frequency. For the latter, geometry relaxations along the reaction coordinate were also carried out to confirm that they connect the corresponding reaction energy minima.

### Synthesis of Metal Complexes

#### Synthesis of (^Mes^CCC)Fe(SiH_2_Ph)(Py)(N_2_) (**2-SiH**_**2**_**Ph**)

A 20 mL scintillation vial equipped with a stir bar was charged with (^Mes^CCC)FeMes(Py) (0.050 g, 0.063 mmol, 1.0 equiv) and hexanes (3 mL). While stirring, PhSiH_3_ (0.008 g, 0.08 mmol, 1.2 equiv) was added via syringe, resulting in a color change to orange red. The suspension was stirred for 1 h, followed by filtration over Celite. The orange precipitate was extracted in benzene and lyophilized to give the product as an orange solid in good yield (0.043 g, 0.055 mmol, 87%). Additional material can be obtained by concentration of the hexane filtrate and recrystallization at −35 °C. Crystals suitable for X-ray diffraction were grown from a concentrated Et_2_O solution of the product at −35 °C. Anal. Calcd for C_49_H_45_FeN_7_Si: C, 72.14; H, 5.56; N, 12.02. Found: C, 72.32; H, 5.62; N, 11.68. NMR data (C_6_D_6_, 25 °C): ^1^H δ = 7.77 (d, *J* = 8.0 Hz, 2H), 7.71–7.64 (m 4H), 7.53 (t, *J* = 7.7 Hz, 1H), 7.07–7.02 (m, 3H), 6.97 (s, 2H), 6.94 (t, *J* = 7.1 Hz, 1H), 6.89 (t, *J* = 7.5 Hz, 2H), 6.82–6.74 (m, 5H), 6.68 (s, 2H), 6.58 (d, *J* = 7.8 Hz, 2H), 6.28 (t, *J* = 7.5 Hz, 1H), 5.78 (d, *J* = 7.0 Hz, 2H), 3.96 (t, 2H), 2.64 (s, 6H), 2.09 (s, 6H), 0.77 (s, 6H). ^13^C{^1^H} δ = 223.70, 181.81, 153.53, 148.70, 143.89, 138.96, 138.86, 138.75, 136.91, 135.05, 134.41, 133.13, 133.07, 130.26, 128.61, 128.25, 126.40, 125.98, 123.28, 121.96, 121.00, 110.14, 108.62, 107.61, 21.16, 19.77, 16.47. ^29^Si{^1^H} δ = 10.87. ATR-IR was 2075 cm^–1^ (Fe–N_2_).

#### Synthesis of (^Mes^CCC)Fe(SiHPh_2_)(Py)(N_2_) (**2-SiHPh**_**2**_)

A 20 mL scintillation vial equipped with a stir bar was charged with (^Mes^CCC)FeMes(Py) (0.050 g, 0.063 mmol, 1.0 equiv) and hexanes (3 mL). While stirring, Ph_2_SiH_2_ (0.014 g, 0.076 mmol, 1.1 equiv) was added via syringe, resulting in an immediate color change to orange red. The suspension was stirred for 15 min, followed by filtration over Celite. The orange precipitate was extracted in benzene and lyophilized to give the product as an orange solid in good yield (0.054 g, 0.061 mmol, 97%). Crystals suitable for X-ray diffraction were grown from a concentrated toluene solution of the product with 3 drops of hexanes at −35 °C. Anal. Calcd for C_55_H_49_FeN_7_Si: C, 74.06; H, 5.54; N, 10.99. Found: C, 73.75; H, 5.74; N, 10.78. NMR data (C_6_D_6_, 25 °C): ^1^H δ = 7.77 (d, *J* = 8.1 Hz, 2H), 7.70–7.62 (m, 4H), 7.54 (t, *J* = 7.7 Hz, 1H), 7.23 (d, *J* = 6.7 Hz, 4H), 7.04 (t, *J* = 7.7 Hz, 2H), 6.98–6.92 (m, 4H), 6.86 (q, *J* = 7.0 Hz, 6H), 6.66 (s, 2H), 6.51 (d, *J* = 7.9 Hz, 2H), 6.26 (t, *J* = 7.6 Hz, 1H), 5.79 (t, *J* = 6.7 Hz, 2H), 4.45 (s, 1H), 2.32 (s, 6H), 2.09 (s, 6H), 0.69 (s, 6H). ^13^C{1H} δ = 224.47, 183.25, 153.37, 149.19, 146.58, 139.12, 138.91, 138.79, 137.05, 135.75, 134.41, 133.28, 133.07, 130.05, 126.31, 126.26, 123.46, 121.94, 121.36, 110.25, 108.82, 107.78, 21.16, 19.92, 16.51. ^29^Si{1H} δ = 28.22. ATR-IR = 2070 cm^–1^ (Fe–N2).

#### Synthesis of (^Mes^CCC)Fe(SiMe_2_Ph)(Py)(N_2_) (**2-SiMe**_**2**_**Ph**)

A 20 mL scintillation vial with a stir bar was charged with (^Mes^CCC)FeMes(Py) (0.050 g, 0.063 mmol, 1.0 equiv) and THF (4 mL). While stirring, Me_2_PhSiH (0.010 g, 0.076 mmol, 1.1 equiv) was added via syringe, and the solution was stirred overnight, resulting in a color change to orange red. After stirring, the solution was filtered over Celite, and volatiles were removed under reduced pressure. The orange residue was washed with Et_2_O (2× 2 mL) and extracted in C_6_H_6_. Removal of volatiles under reduced pressure afforded the product as an orange powder in a good yield (0.048 g, 0.057 mmol, 91%). Additional material can be obtained from the filtrate by crystallization at −35 °C. Crystals suitable for X-ray diffraction were grown from a solution of the product in HMDSO and pentane at −35 °C. Anal. Calcd for C_51_H_49_FeN_7_Si: C, 72.58; H, 5.85; N, 11.62. Found: C, 72.69; H, 6.41; N, 11.41. NMR data (C_6_D_6_, 25 °C): ^1^H δ = 7.74–7.70 (m, 4H), 7.60 (t, *J* = 7.7 Hz, 1H), 7.01 (t, *J* = 7.6 Hz, 2H), 6.95 (s, 2H), 6.87 (t, *J* = 7.6 Hz, 3H), 6.83–6.76 (m, 4H), 6.56–6.46 (m, 4H), 6.27 (t, *J* = 7.5 Hz, 1H), 5.77 (br, 2H), 2.72 (s, 6H), 2.01 (s, 6H), 0.41 (s, 6H), 0.24 (s, 6H). ^13^C{^1^H} δ = δ 225.52, 183.41, 151.02, 149.11, 139.26, 138.65, 138.22, 137.59, 134.55, 133.95, 132.96, 132.73, 129.85, 125.88, 125.50, 123.34, 121.85, 121.74, 120.39, 110.18, 108.50, 107.53, 21.09, 19.98, 15.89, 4.12. ^29^Si{^1^H} δ = 21.83. ATR-IR = 2061 cm^–1^ (Fe–N_2_).

#### Synthesis of (^Mes^CCC)Fe[SiMe(OSiMe_3_)_2_](Py)(N_2_) (**2-MD’M**)

A 20 mL scintillation vial equipped with a stir bar was charged with (^Mes^CCC)FeMes(Py) (0.050 g, 0.063 mmol, 1.0 equiv) and THF (4 mL). While stirring, MD’M (0.017 g, 0.076 mmol, 1.1 equiv) was added via syringe, and the solution was stirred for 4 h, resulting in a color change to orange–red. After stirring, the suspension was filtered over Celite, and volatiles were removed under reduced pressure. The orange residue was washed with HMDSO (2× 2 mL) and extracted in benzene. Removal of volatiles under reduced pressure afforded the product as an orange powder in good yield (0.049 g, 0.053 mmol, 84%). Crystals suitable for X-ray diffraction were grown from a solution of the product in HMDSO and Et_2_O at −35 °C. Anal. Calcd for C_50_H_59_FeN_7_O_2_Si_3_: C, 64.56; H, 6.39; N, 10.54. Found: C, 64.29; H, 6.68; N, 10.17. NMR data (C_6_D_6_, 25 °C): ^1^H δ = 7.99 (d, *J* = 8.0 Hz, 2H), 7.84 (d, *J* = 7.7 Hz, 2H), 7.60 (t, *J* = 7.7 Hz, 1H), 7.09 (t, *J* = 7.6 Hz, 2H), 7.04 (s, 2H), 6.92 (t, *J* = 7.6 Hz, 2H), 6.66 (s, 2H), 6.57 (d, *J* = 7.9 Hz, 2H), 6.24 (t, *J* = 7.5 Hz, 1H), 5.75 (s, 2H), 2.66 (s, 6H), 2.10 (s, 6H), 0.65 (s, 6H), 0.33 to −0.32 (m, 18H), −0.45 (s, 3H). ^13^C{^1^H} δ = 184.99, 153.08, 149.38, 138.82, 138.68, 136.75, 134.38, 132.96, 132.72, 129.76, 123.38, 121.92, 121.87, 120.29, 110.13, 108.74, 21.20, 20.51, 16.34, 8.11, 3.35, 0.80. ^29^Si{^1^H} δ = 28.98, −1.32. ATR-IR = 2084 cm^–1^ (Fe–N_2_).

## Supporting Information Available

Experimental spectra, computational details, and crystallographic information are available in the Supporting Information and on the Cambridge Structural Database: CCDC 2363644–236364523636462363647. Additionally, all the DFT-optimized structures reported in this work are openly accessible from the ioChem-BD repository available at the following link: https://iochem-bd.bsc.es/browse/review-collection/100/321082/f6549ec31c17a9cbd2257c1f. NMR spectra of metal complexes, reactivity studies, catalytic hydrogenation of olefins, crystallographic parameters, and computational methods (PDF)
