# Supplementary material for: Identity of the Silyl Ligand in an Iron Silyl Complex Influences Olefin Hydrogenation: An Experimental and Computational Study
Source: Inorg Chem. 2024 Sep 10;63(38):17706–13. doi: 10.1021/acs.inorgchem.4c02533 (PMC11423403; doi:10.1021/acs.inorgchem.4c02533)
Supplement: Supplementary file 1 — ic4c02533_si_001.pdf [file ic4c02533_si_001.pdf]

Identity of the Silyl Ligand in an Iron-Silyl Complex Influences Olefin Hydrogenation: An Experimental and Computational Study

Daniel C. Najera,[a] *Marconi N. Peñas-Defrutos,*[b,c*] *Max García-Melchor,*[b*] *Alison R. Fout*[d*]

[a] School of Chemical Sciences, University of Illinois at Urbana-Champaign, 600 S. Mathews Ave., Urbana, Illinois 61801, USA.

[b] School of Chemistry, CRANN and AMBER Research Centres, Trinity College Dublin, College Green, Dublin 2, Ireland.

[c] IU CINQUIMA/Química Inorgánica, Facultad de Ciencias, Universidad de Valladolid, 47071-Valladolid, Spain.

[d] Department of Chemistry, Texas A&M University, College Station, TX 77840.

Supporting Information Table of Contents:

NMR Spectra of Metal Complexes..1
Reactivity Studies..7
Catalytic Hydrogenation of Olefins..10
Crystallographic Parameters..14
Computational Methods..15

NMR Spectra of Metal Complexes

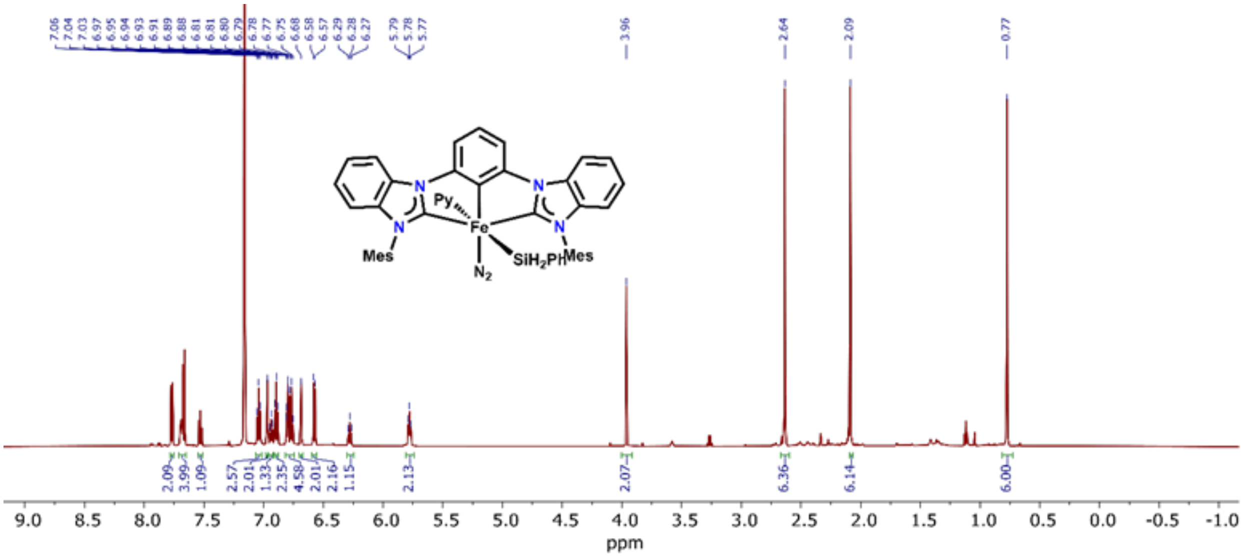

Figure S1. ^{1}H NMR spectrum of **2-SiH$_2$Ph** in C_6D_6.

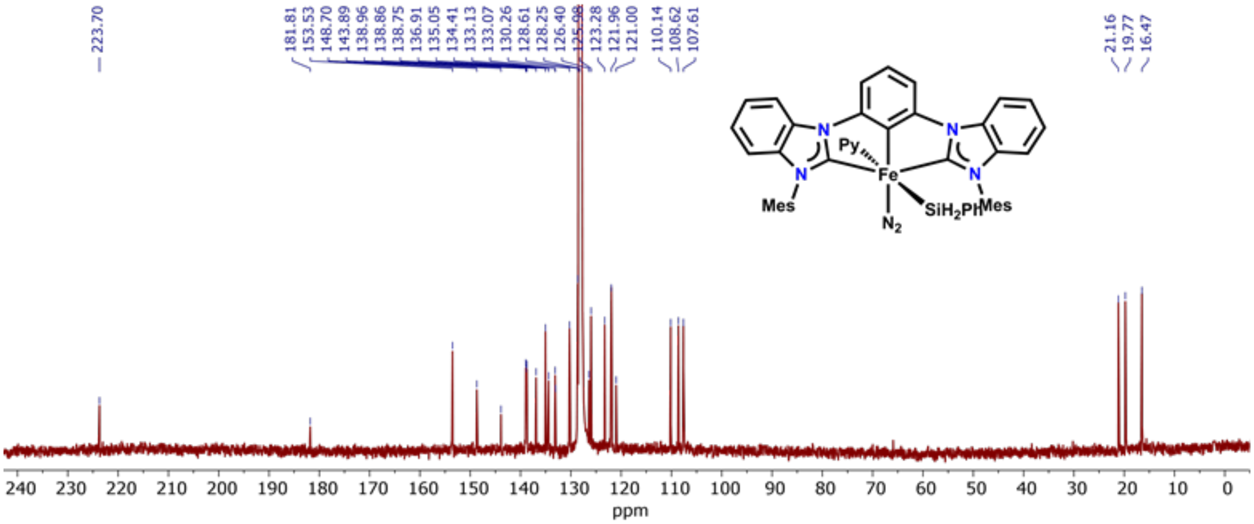

Figure S2. $^{13}C\{^1H\}$ NMR spectrum of **2-SiH$_2$Ph** in C_6D_6.

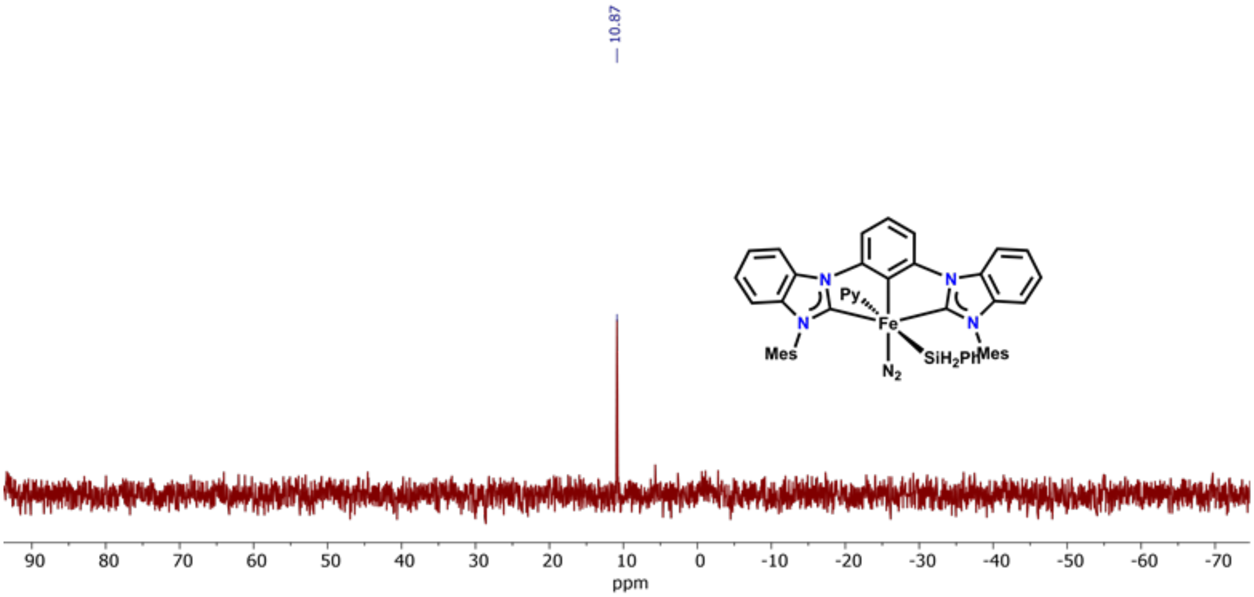

Figure S3. $^{29}Si\{^{1}H\}$ NMR spectrum of **2-SiH₂Ph** in C_6D_6.

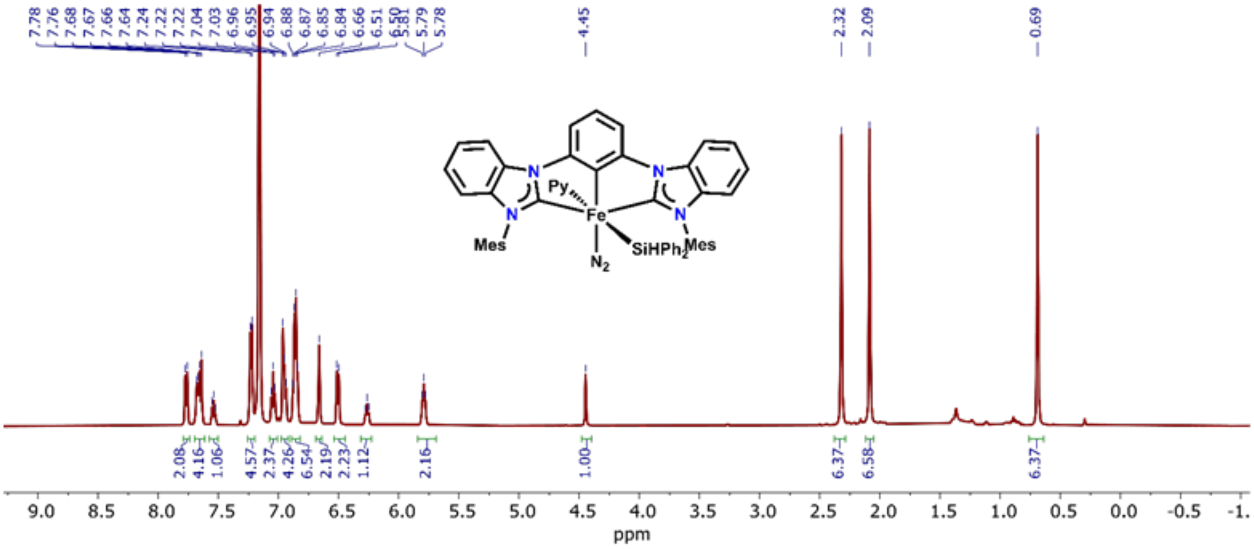

Figure S4. ^{1}H NMR spectrum of **2-SiHPh₂** in C_6D_6.

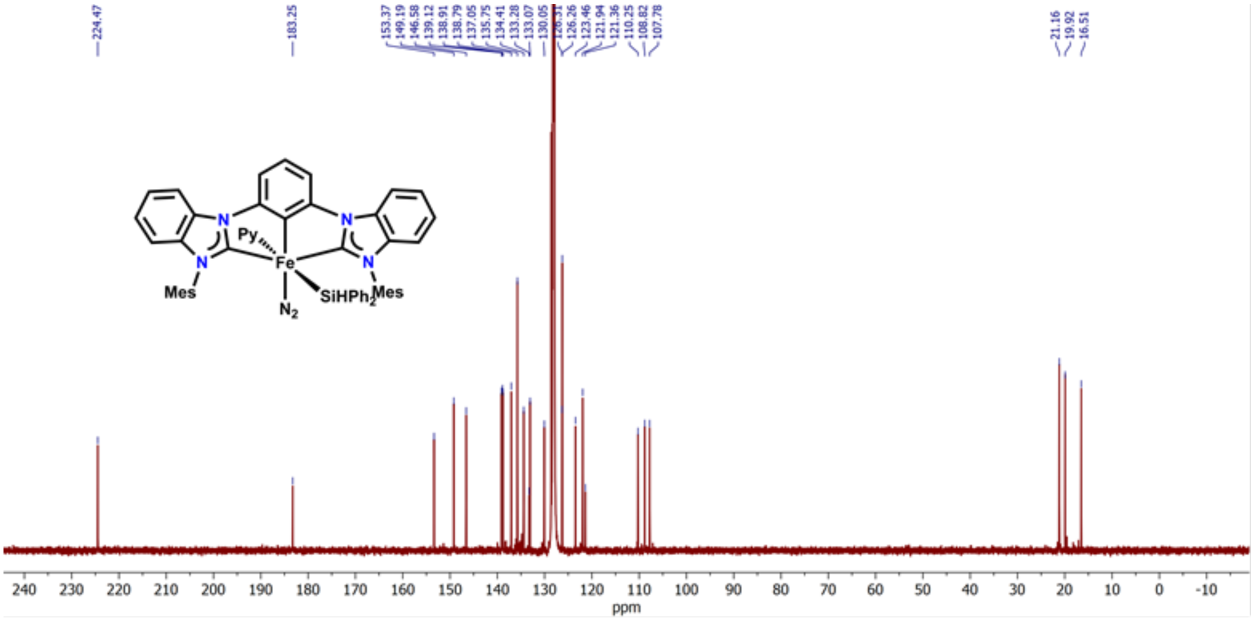

Figure S5. $^{13}C\{^{1}H\}$ NMR spectrum of **2-SiHPh₂** in C_6D_6.

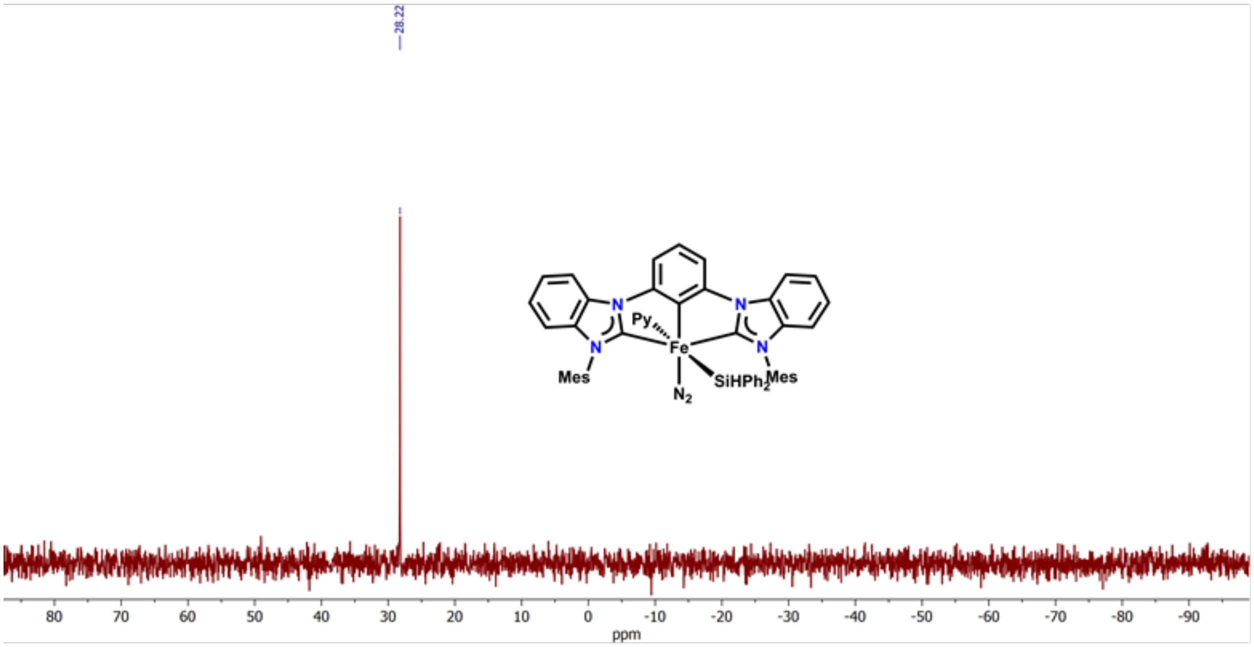

Figure S6. $^{29}Si\{^{1}H\}$ NMR spectrum of **2-SiHPh₂** in C_6D_6.

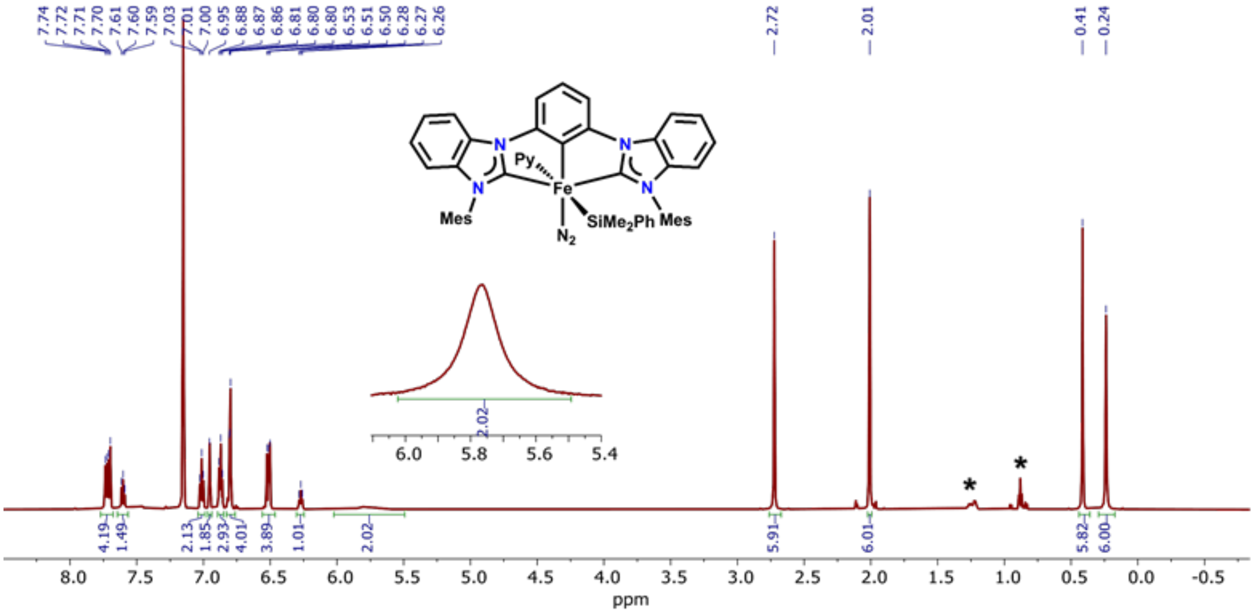

Figure S7. ^{1}H NMR spectrum of **2-SiMe₂Ph** in C_6D_6. (*) Denotes hexane.

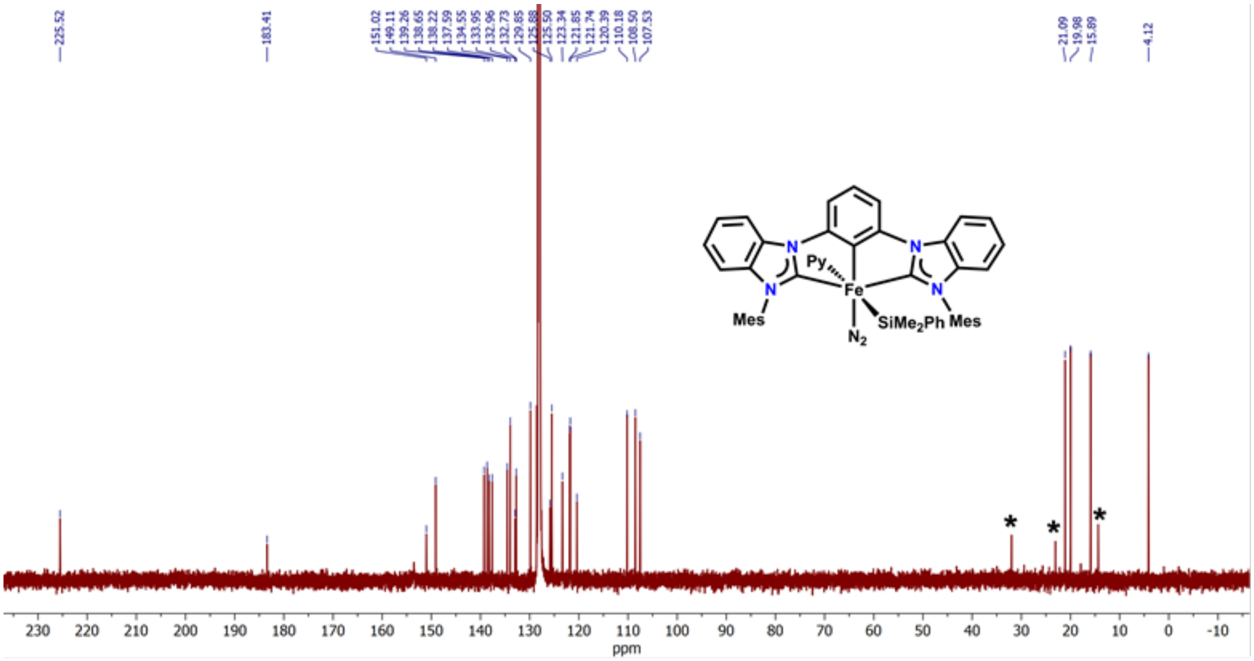

Figure S8. $^{13}C\{^{1}H\}$ NMR spectrum of **2-SiMe₂Ph** in C_6D_6. (*) Denotes hexane.

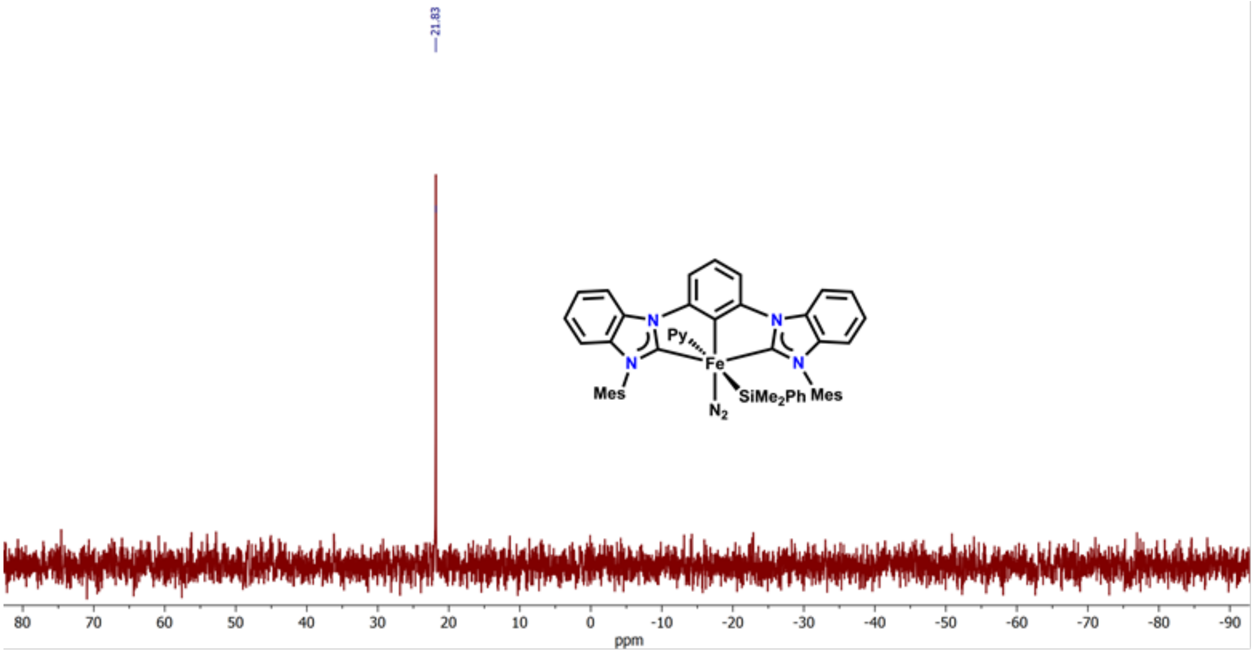

Figure S9. $^{29}Si\{^{1}H\}$ NMR spectrum of **2-SiMe2Ph** in C_6D_6.

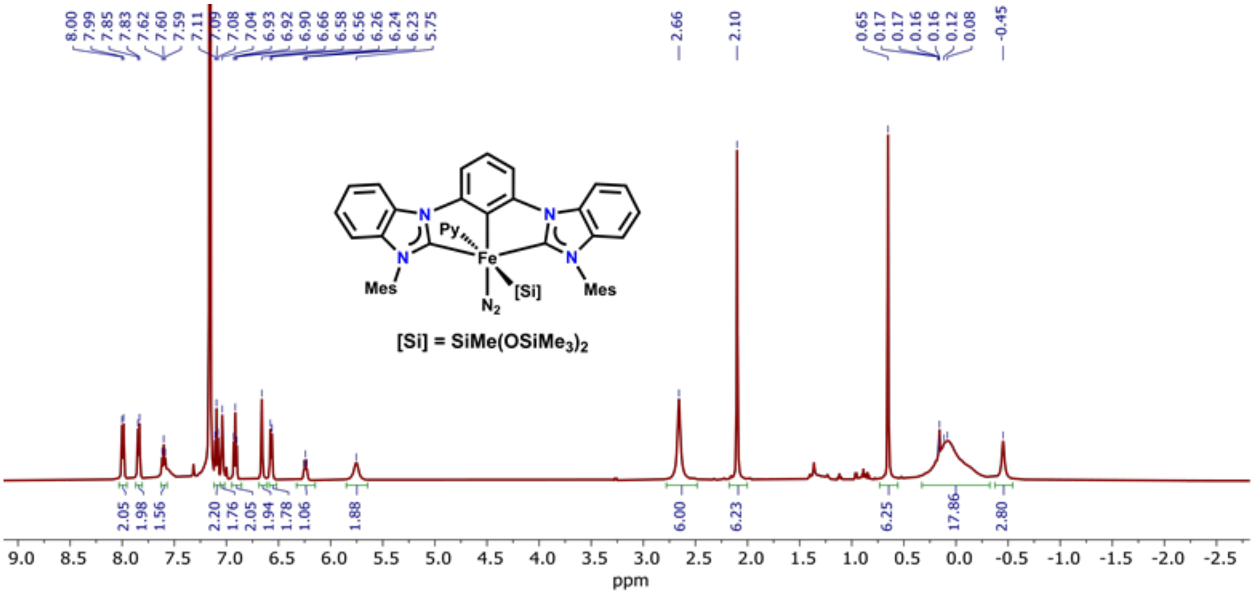

Figure S10. ^{1}H NMR spectrum of **2-MD'M** in C_6D_6.

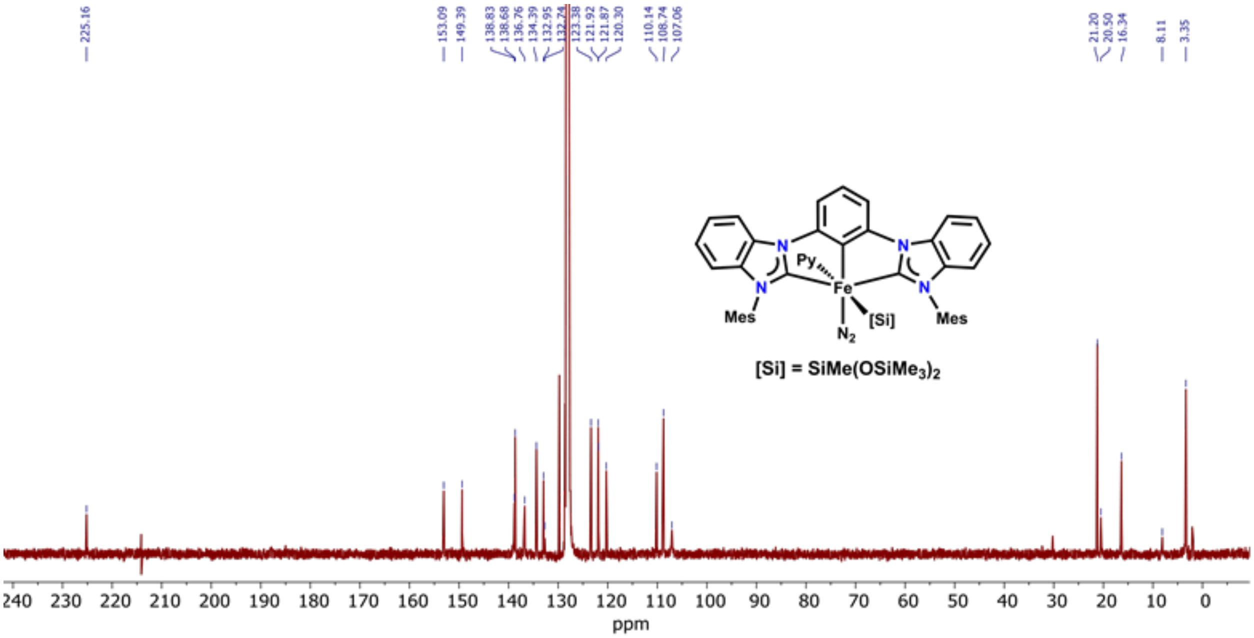

Figure S11. $^{13}C\{^1H\}$ NMR spectrum of **2-MD'M** in C_6D_6.

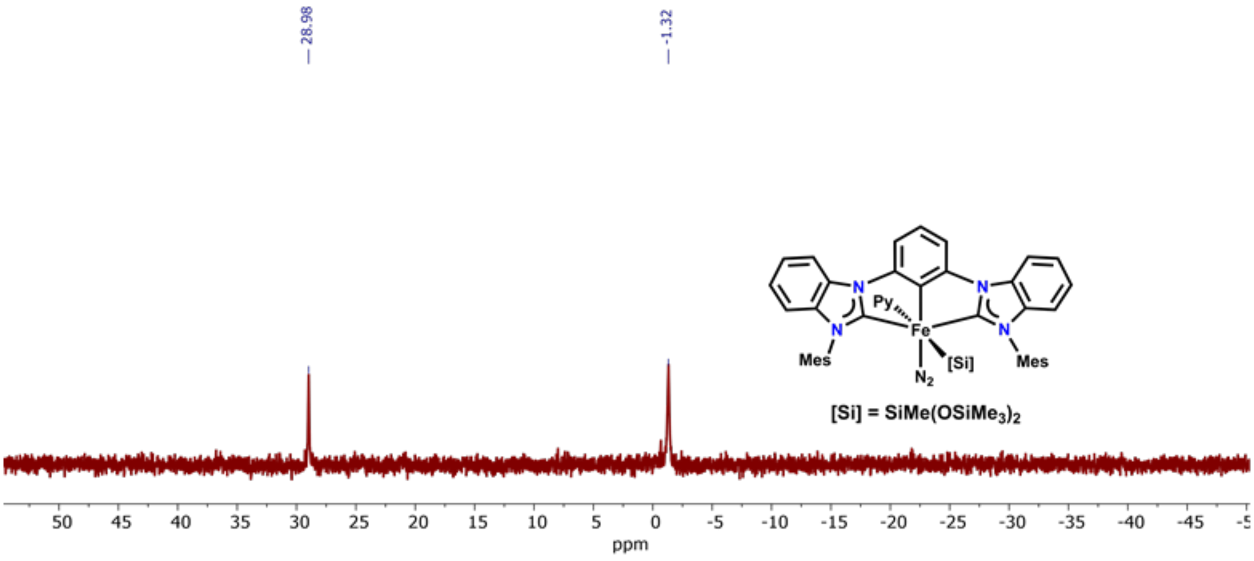

Figure S12. $^{29}Si\{^1H\}$ NMR spectrum of **2-MD'M** in C_6D_6.

Reactivity Studies

Reaction of 2-Si with H_2 and D_2. To a 4 mL scintillation vial was added **2-Si** (0.010 mmol) and THF-d_8 (ca. 0.7 mL). The solution was transferred to an NMR tube fitted with a J. Young valve, sealed, and removed from the glovebox. The sample was subjected to two freeze-pump-thaw cycles. On a third pump step, 1 atm of H_2 or D_2 was added to the sample at 77 K, resulting in 4 atm at room temperature. The sample was analyzed by ^{1}H or ^{2}H NMR spectroscopy.

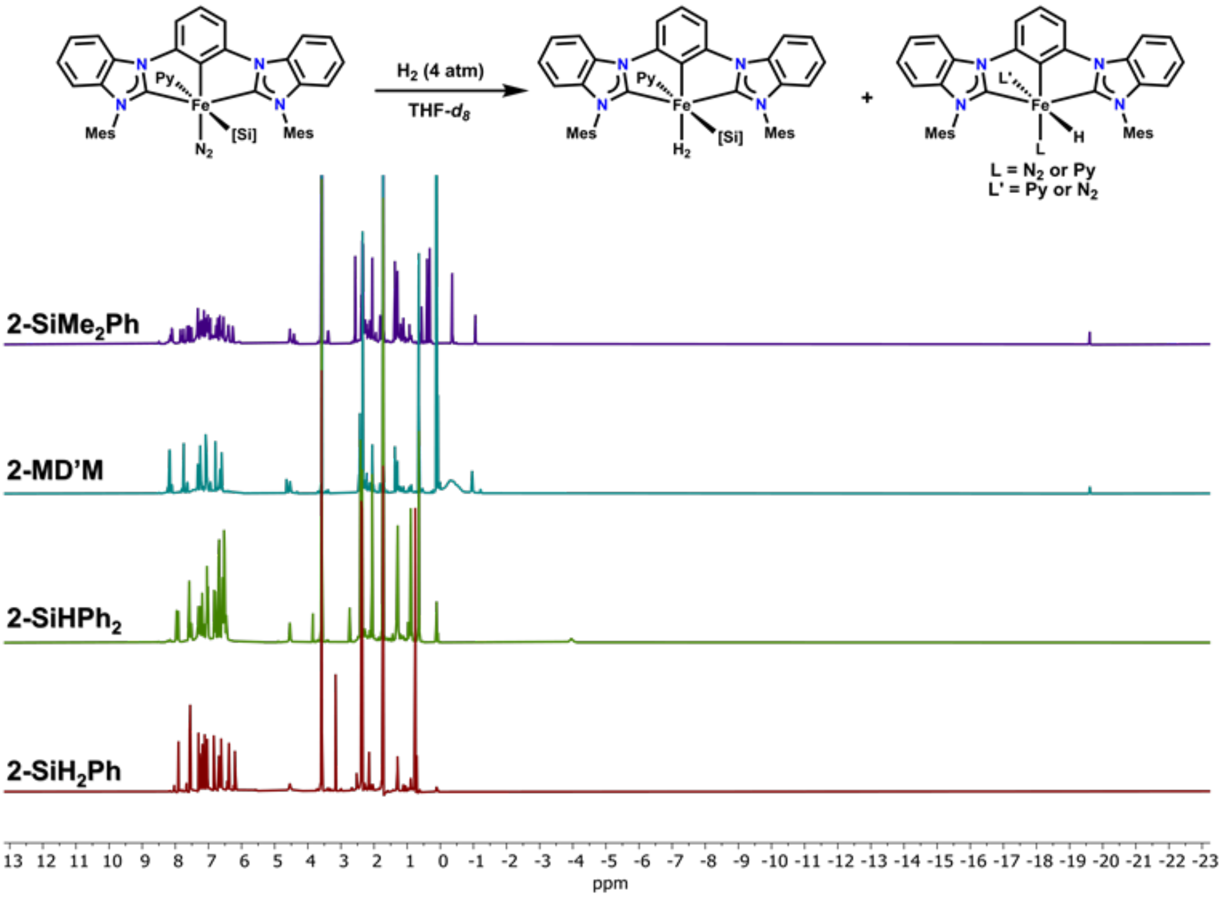

Figure S13. Stacked ^{1}H NMR spectra of the reaction between **2-Si** and H_2 in THF-d_8.

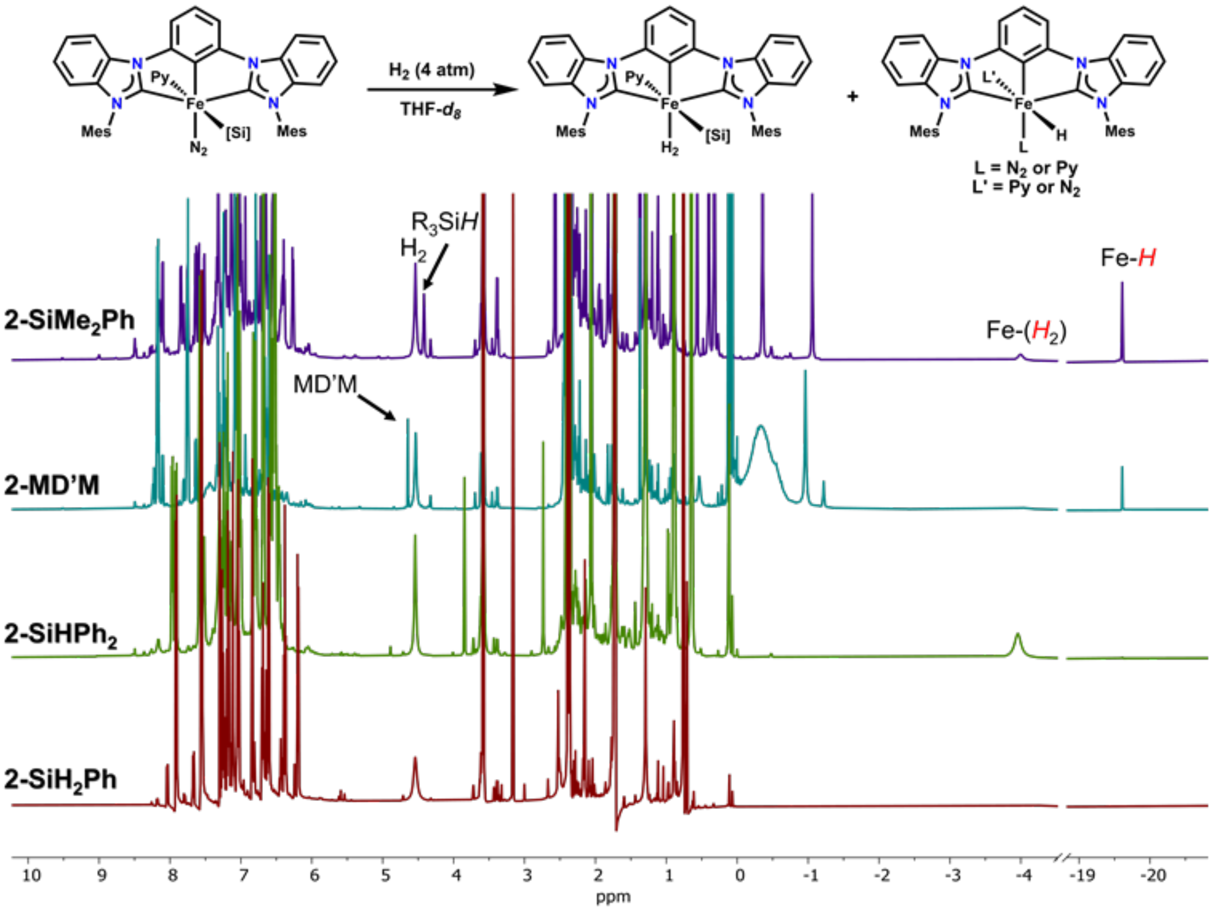

Figure S14. Stacked ^{1}H NMR spectra of the reaction between **2-Si** and H_2 in THF-d_8, amplified to show formation of Me_2PhSiH and MD'M.

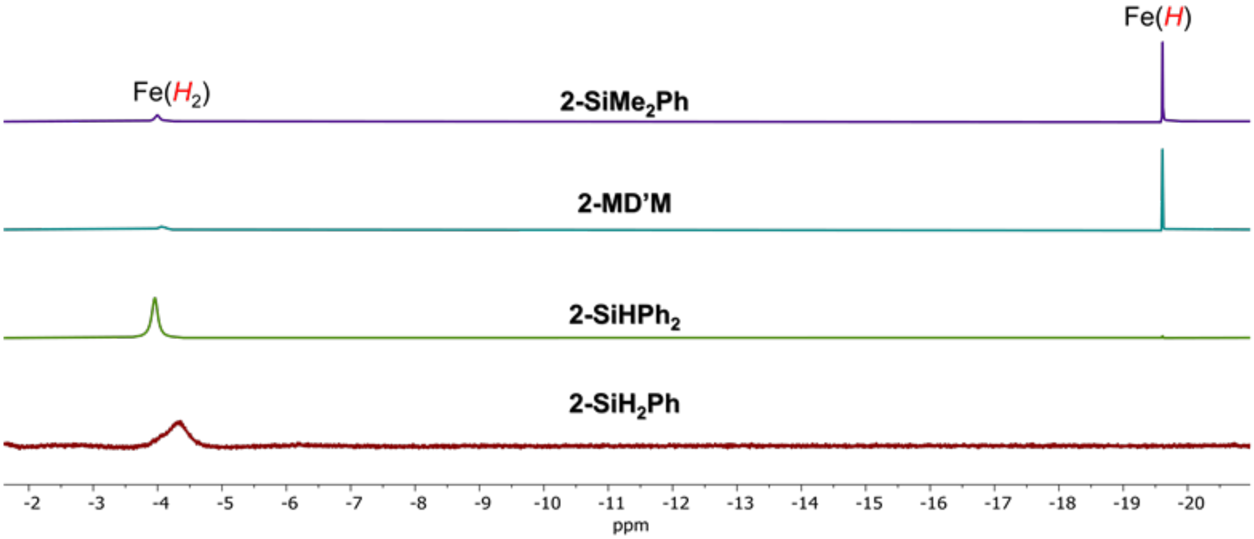

Figure S15. Upfield region of the ^{1}H NMR spectra of the reaction between **2-Si** and H_2.

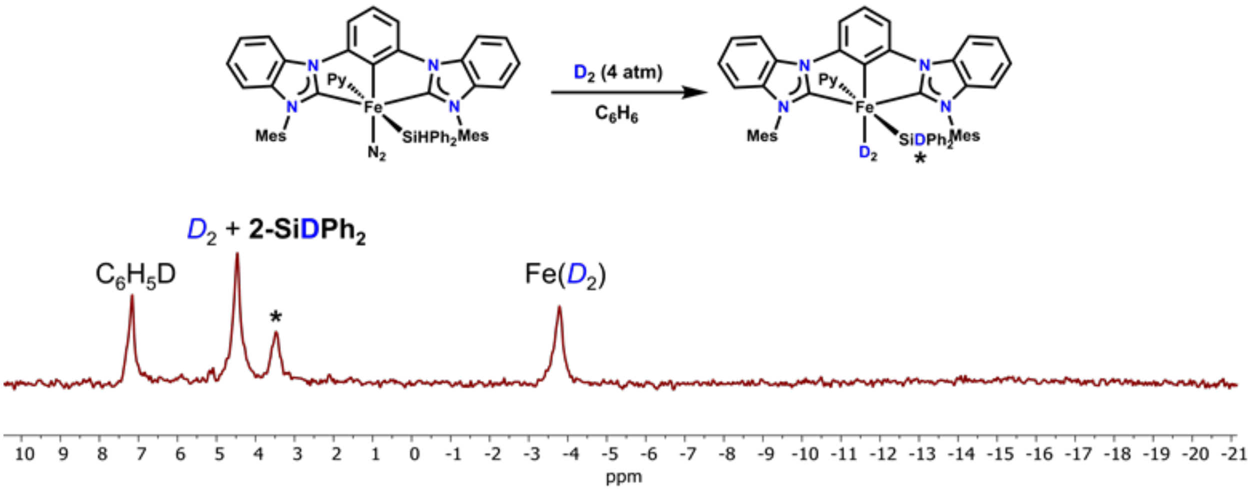

Figure S16. ${}^{2}H$ NMR spectrum of the reaction between **2-SiHPh$_2$** and D_2 in C_6H_6. (*) Denotes the deuterium atom on the silyl ligand of the product.

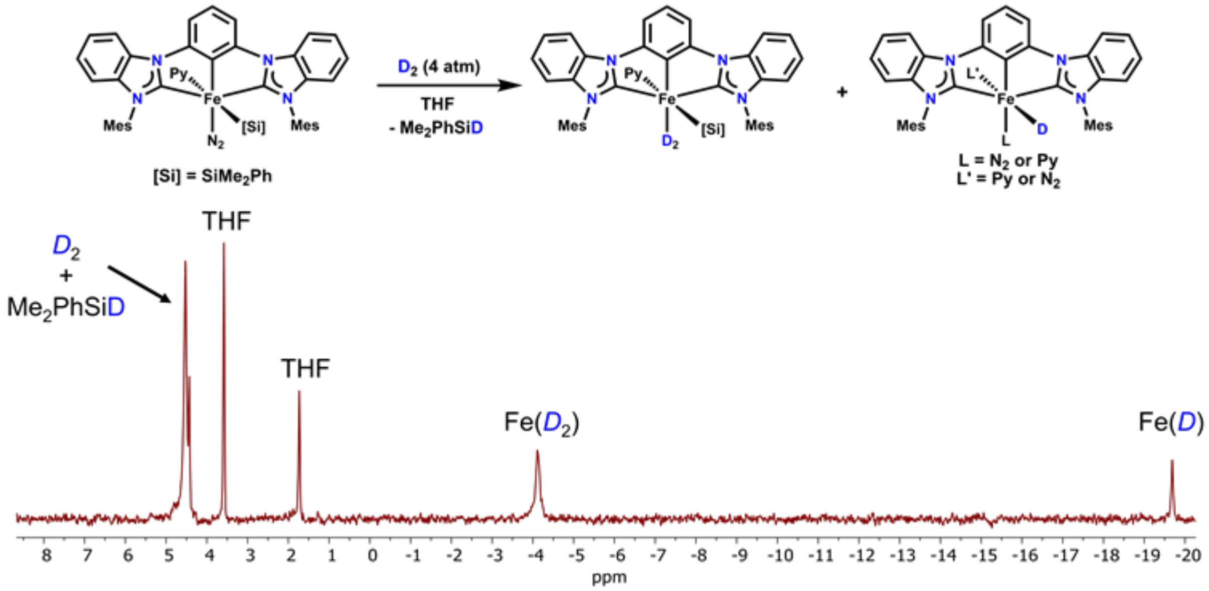

Figure S17. ${}^{2}H$ NMR spectrum of the reaction between S-SiMe$_2$Ph and D_2 in THF.

HD scrambling by 2-SiH$_2$Ph and 2-SiHPh$_2$. To a 4 mL scintillation vial, **2-Si** (0.010 mmol) and C_6D_6 or THF-d_8 (ca. 0.7 mL) were added. The solution was transferred to an NMR tube fitted with a J. Young valve, sealed, and removed from the glovebox. The sample was subjected to two freeze-pump-thaw cycles. On a third pump step, 1 atm of a 1:1 mixture of H_2 and D_2 was added to the sample at 77 K, resulting in 4 atm at room temperature. The sample was analyzed by ${}^{1}H$ NMR.

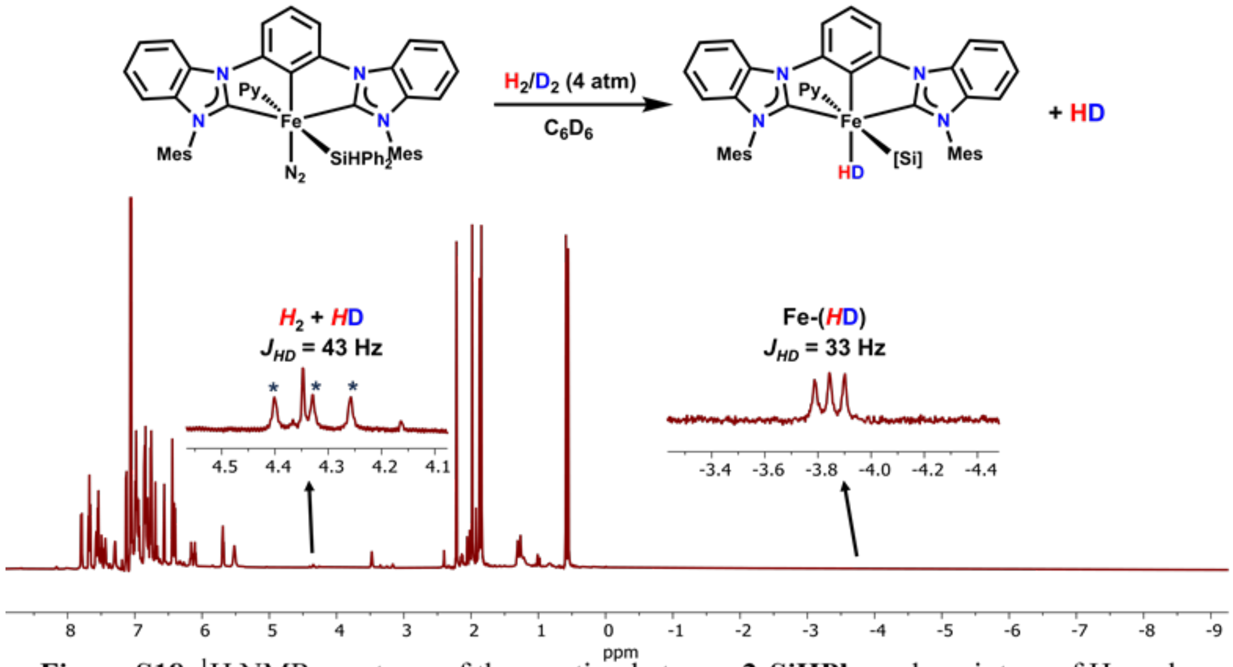

Figure S18. ^{1}H NMR spectrum of the reaction between **2-SiHPh₂** and a mixture of H_2 and D_2 in C_6D_6.

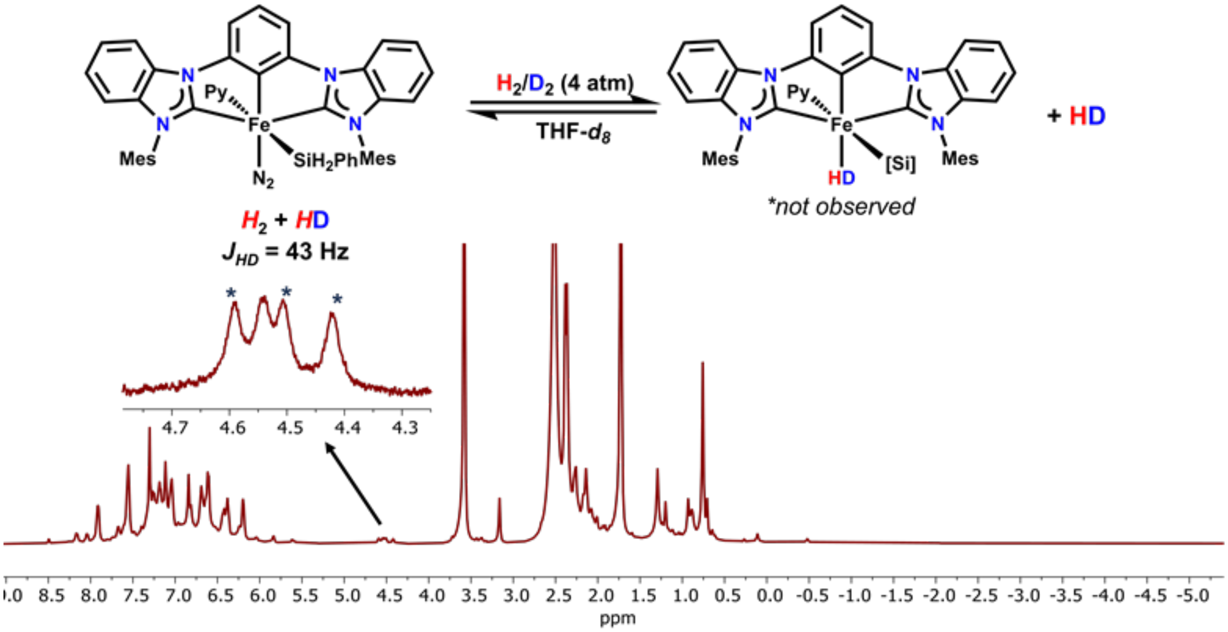

Figure S19. ^{1}H NMR spectrum of the reaction between **2-SiH₂Ph** and a mixture of H_2 and D_2 in THF-d_8.

Catalytic Hydrogenation of Olefins

General hydrogenation procedure. To a 4 mL scintillation vial, **2-Si** (0.0029 mmol, 2 mol%), mesitylene (0.144 mmol, 1 equiv.), substrate (0.144 mmol, 1 equiv.) and 0.5 mL of THF-d_8 were added. The mixture was transferred to a J. Young NMR tube and taken out of the glovebox. The sample was subjected to two freeze-pump-thaw cycles. After a third pump stage, 1 atm of H_2 was

added at 77 K resulting in 4 atm of H_2 at room temperature. After thawing, the sample was secured with Parafilm to a 14/20 solvent trap connected to a rotary evaporator and subjected to repeated inversion on the lowest rotation setting. Conversion to the product was monitored by ^1H NMR spectroscopy.

^1H NMR spectra of catalytic hydrogenation reactions

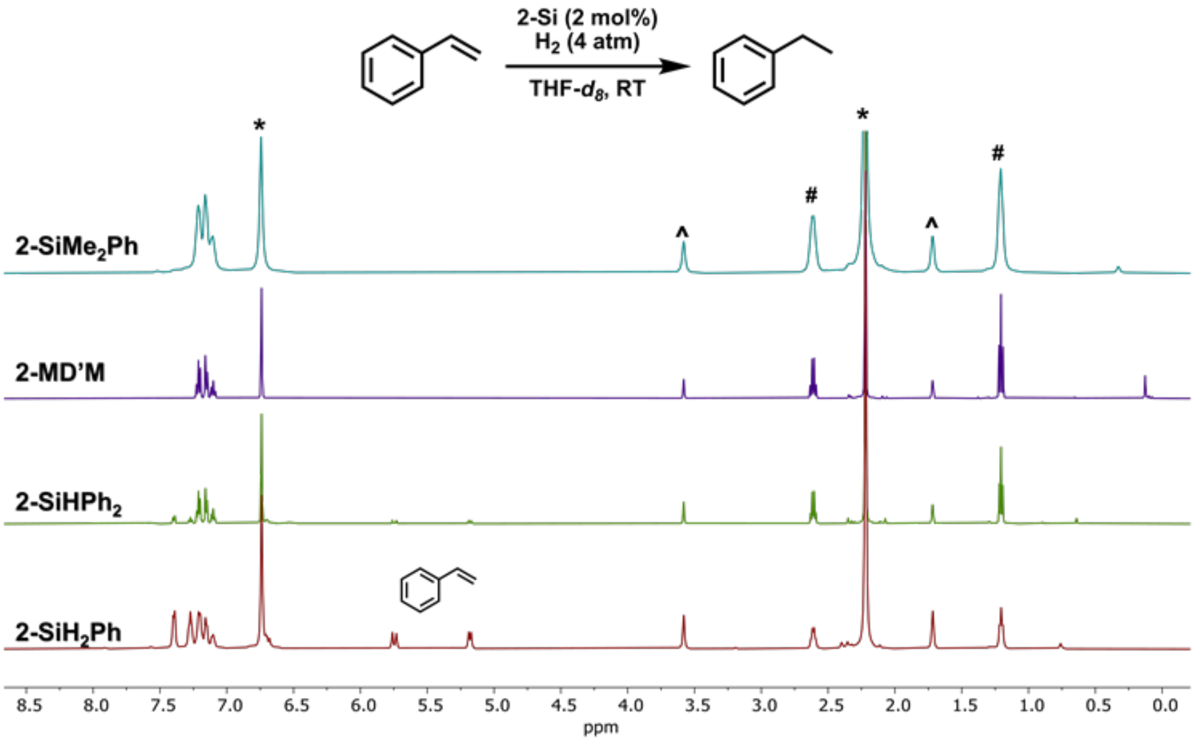

Figure S20. ^1H NMR spectra of the hydrogenation of styrene with **2-Si**, and 4 atm of H_2 in THF-d_8 after 1 hour. (#) Denotes ethylbenzene, (*) denotes mesitylene, (^) denotes THF.

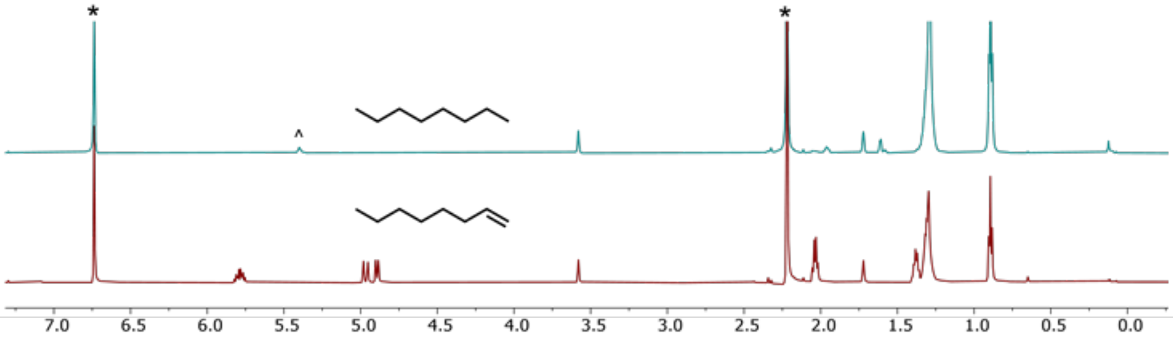

Figure S21. ^{1}H NMR spectra of **2-MD'M**, 1-octene, and mesitylene prior to H_2 addition (bottom) and after 2 h under 4 atm of H_2 (top) in THF-d_8. (*) Denotes mesitylene, (^) denotes internal alkene isomerization product.

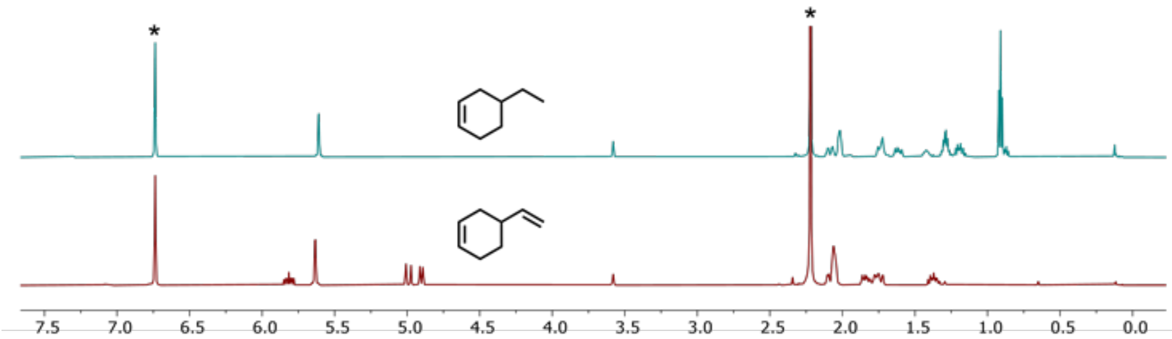

Figure S22. ^{1}H NMR spectra of **2-MD'M**, 4-vinylcyclohexene, and mesitylene prior to H_2 addition (bottom) and after 2 h under 4 atm of H_2 (top) in THF-d_8. (*) Denotes mesitylene.

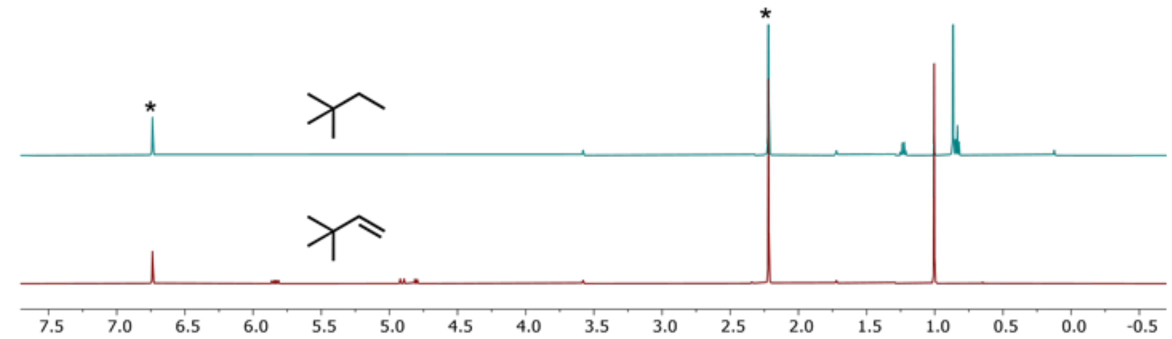

Figure S23. ^{1}H NMR spectra of **2-MD'M**, 3,3-dimethylbutene, and mesitylene prior to H_2 addition (bottom) and after 40 h under 4 atm of H_2 (top) in THF-d_8. (*) Denotes mesitylene.

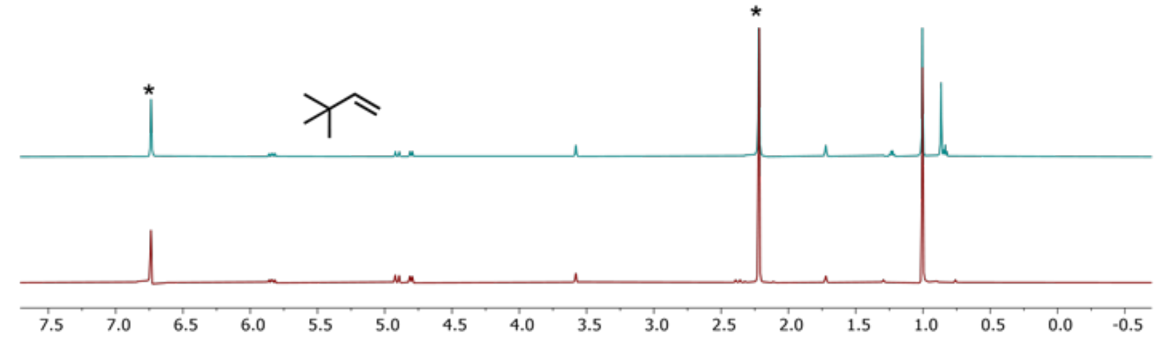

Figure S24. ^{1}H NMR spectra of **2-SiH$_2$Ph**, 3,3-dimethylbutene, and mesitylene prior to H_2 addition (bottom) and after 40 h under 4 atm of H_2 (top) in THF-d_8. (*) Denotes mesitylene.

Crystallographic Parameters

Table S1. Crystallographic Parameters of Complexes **2-Si**

	**2-SiH$_2$Ph**	**2-SiHPh$_2$**	**2-SiMe$_2$Ph**	**2-MD'M**
Empirical formula	$C_{49}H_{45}FeN_7Si$	$C_{55}H_{49}FeN_7Si$	$C_{51}H_{49}FeN_7Si$	$C_{50}H_{59}FeN_7O_2Si_3$
Formula weight	815.86	891.95	843.91	930.16
Temperature	100.00	100.0	100.00	100.00
Crystal system	Monoclinic	Triclinic	Orthorhombic	Monoclinic
Space group	P2(1)/c	P-1	Pbca	P2$_1$/n
Unit Cell Dimensions	a = 22.0201(12) Å	a = 10.2530(3) Å	a = 16.5099(4) Å	a = 11.2527(2) Å
	b = 8.3125(4) Å	b = 22.2465(6) Å	b = 21.2214(5) Å	b = 24.7062(5) Å
	c = 22.3466(12) Å	c = 23.8698(7) Å	c = 24.5338(5) Å	c = 17.3814(3) Å
	α = 90°	α = 76.6890(10)°	α = 90°	α = 90°
	β = 97.197(2)°	β = 84.7350(10)°	β = 90°	β = 91.0450(10)°
	γ = 90°	γ = 89.4350(10)°	γ = 90°	γ = 90°
Volume	4058.1(4) Å^3	5275.6(3) Å^3	8595.7(3) Å^3	4831.42(15) Å^3
Z	4	4	8	4
Reflections collected	66475	406080	165389	179877
Independent reflections	66475<br>R_{int} = 0.0976<br>R_{sigma} = 0.0620	26233<br>R_{int} = 0.0490<br>R_{sigma} = 0.0179	7897<br>R_{int} = 0.0685<br>R_{sigma} = 0.0192	12025<br>0.0650<br>0.0242
Goodness-of-fit on F^2	1.012	1.031	1.039	1.061
Final R indexes [I>=2σ (I)]	R_1 = 0.0724<br>wR_2 = 0.1551	R_1 = 0.0325<br>wR_2 = 0.0827	R_1 = 0.0375<br>wR_2 = 0.0920	R_1 = 0.0328<br>wR_2 = 0.0848

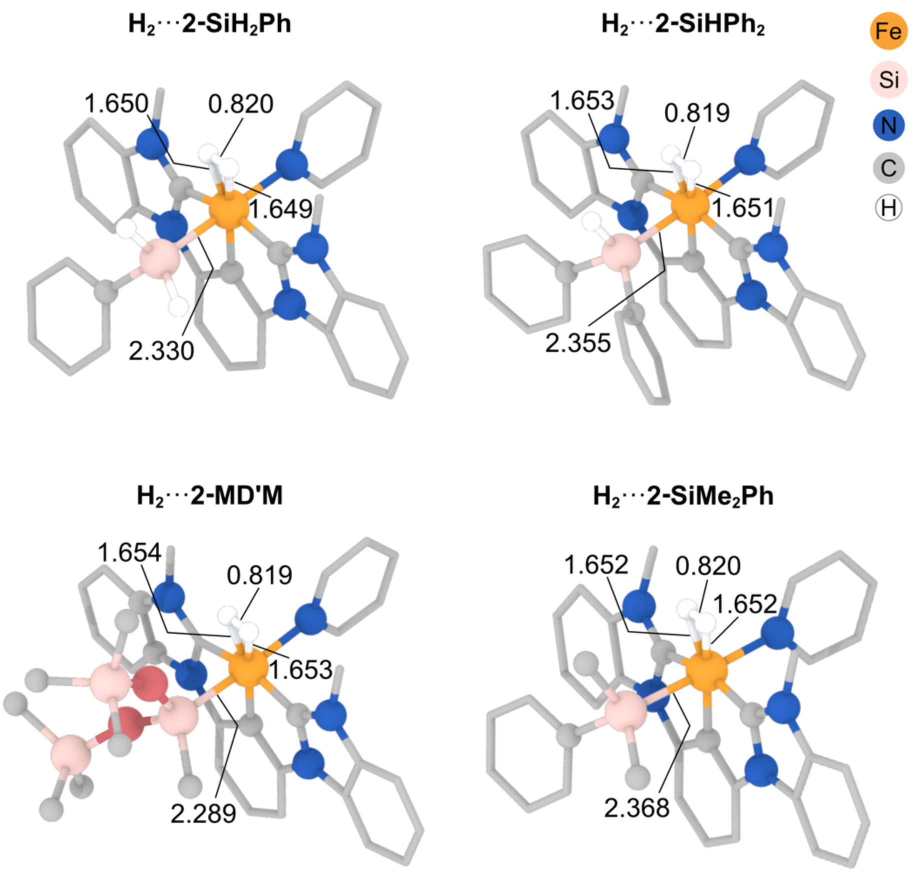

Figure S25. Optimized structures of the (^{Mes}CCC)Fe(SiR^{1}R^{2}R^3)(Py)(H_2) complexes (**H_2···2-Si**). Most of the H atoms have been omitted for clarity, and the mesityl substituents of the ^{Mes}CCC ligand have been simplified. Relevant distances are shown in Å.

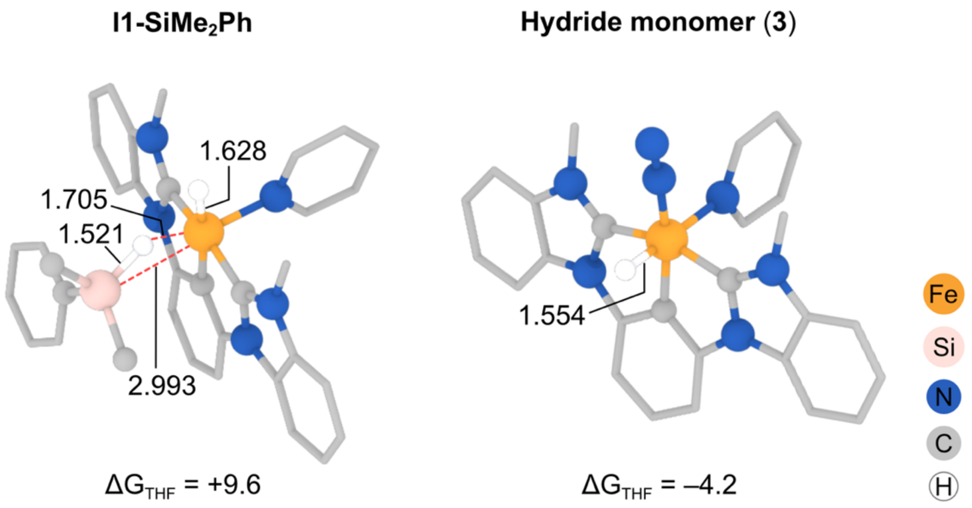

Figure S26. Left: optimized structure of the hydride complex (^{Mes}CCC)FeH(Py)(HSiMe$_2$Ph) obtained by relaxing the geometry of the transition state depicted in Figure 4 along the reaction coordinate. Right: most stable isomer of the hydride monomer **3**. Most of the H atoms have been omitted for clarity, and the mesityl

substituents of the ^{Mes}CCC ligand have been simplified. Gibbs energies (in kcal/mol) calculated in THF relative to **H_2···2-SiMe$_2$Ph** are provided. Relevant distances are shown in Å.

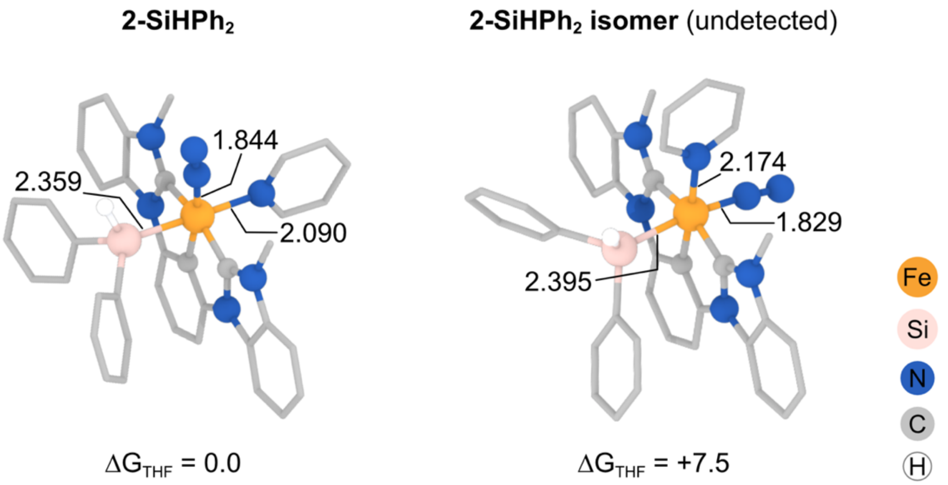

Figure S27. Optimized structures of **2-SiHPh$_2$** (left) and its alternative isomer (right) with the N_2 group in *trans* position with respect to the silane. Most of the H atoms have been omitted for clarity**,** and the mesityl substituents of the ^{Mes}CCC ligand have been simplified. Relative Gibbs energies (in kcal/mol) calculated in THF, and relevant distances (in Å) are shown.

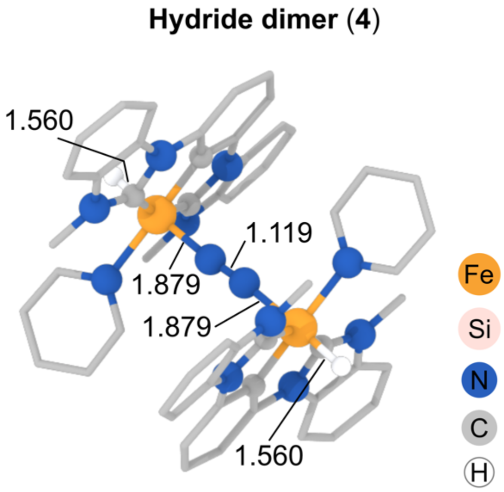

Figure S28. Optimized structure of the lowest energy isomer for the complex (μ-N_2)[(^{Mes}CCC)Fe(H)(Py)]$_2$ (**4**), formed via dimerization of two units of **3** and the dissociation of an N_2 molecule. Most of the H atoms have been omitted for clarity, and the mesityl substituents of the ^{Mes}CCC ligand have been simplified. Relevant distances are shown in Å.
